# Mutation of the Zebrafish Nucleoporin *elys* Sensitizes Tissue Progenitors to Replication Stress

**DOI:** 10.1371/journal.pgen.1000240

**Published:** 2008-10-31

**Authors:** Gangarao Davuluri, Weilong Gong, Shamila Yusuff, Kristin Lorent, Manimegalai Muthumani, Amy C. Dolan, Michael Pack

**Affiliations:** 1 Department of Medicine, University of Pennsylvania School of Medicine, Philadelphia, Pennsylvania, United States of America; 2 Department of Cell & Developmental Biology, University of Pennsylvania School of Medicine, Philadelphia, Pennsylvania, United States of America; European Molecular Biology Laboratory, Germany

## Abstract

The recessive lethal mutation *flotte lotte* (*flo*) disrupts development of the zebrafish digestive system and other tissues. We show that *flo* encodes the ortholog of *Mel-28/Elys*, a highly conserved gene that has been shown to be required for nuclear integrity in worms and nuclear pore complex (NPC) assembly in amphibian and mammalian cells. Maternal *elys* expression sustains zebrafish *flo* mutants to larval stages when cells in proliferative tissues that lack nuclear pores undergo cell cycle arrest and apoptosis. p53 mutation rescues apoptosis in the *flo* retina and optic tectum, but not in the intestine, where the checkpoint kinase Chk2 is activated. Chk2 inhibition and replication stress induced by DNA synthesis inhibitors were lethal to *flo* larvae. By contrast, *flo* mutants were not sensitized to agents that cause DNA double strand breaks, thus showing that loss of Elys disrupts responses to selected replication inhibitors. Elys binds Mcm2-7 complexes derived from Xenopus egg extracts. Mutation of *elys* reduced chromatin binding of Mcm2, but not binding of Mcm3 or Mcm4 in the *flo* intestine. These *in vivo* data indicate a role for Elys in Mcm2-chromatin interactions. Furthermore, they support a recently proposed model in which replication origins licensed by excess Mcm2-7 are required for the survival of human cells exposed to replication stress.

## Author Summary

DNA replication is a complex process that requires activation of cell cycle checkpoints and DNA repair pathways. Genetic analyses in fungi have suggested that nucleoporins, the proteins that make up the nuclear pore complex (NPC), play a role in the cellular response to agents that disrupt cell proliferation or damage DNA. Here we show that mutation of the Elys nucleoporin causes widespread apoptosis in the intestine and other tissues of zebrafish *flotte lotte* (*flo*) mutants. Intestinal apoptosis occurs in the absence of the DNA damage marker γH2X, and levels of chromatin bound Mcm2, a component of the DNA replication helicase, were also reduced in *flo* mutants. These findings suggested that *flo* intestinal cells cannot repair endogenous replication errors. Consistent with this idea, *flo* mutants were highly sensitized to treatment with DNA replication inhibitors such as hydroxyurea, UV irradiation, or cisplatin, but not agents that cause DNA double strand breaks, such as γ-irradiation or camptothecin. These data point to a conserved role for nucleoporins in the cellular response to replication stress in eukaryote cells.

## Introduction

Programmed cell death is believed to function in two contexts during early mammalian development. Prior to implantation and near gastrulation, apoptosis eliminates embryonic cells rendered unfit by growth factor deficiency [1]. At other stages, apoptosis serves a morphogenetic role by eliminating cells required for tissue reorganization [2],[3].

Although apoptosis is normally activated in only a small number of cells of early mammalian embryos, gene targeting experiments have demonstrated the susceptibility of surviving cells. Genomic instability is speculated to be one possible underlying cause of this predisposition, since mutation of DNA repair [4]–[11] and cell cycle checkpoint genes [12],[13] can activate apoptosis of inner cell mass cells. This susceptibility of embryonic progenitor cells to apoptosis persists through later developmental stages as evidenced by the effect of conditional inactivation of DNA repair and checkpoint genes in specialized cells such as neurons [14] and mammary epithelia [15].

*Elys* is a conserved mammalian gene that is required for embryonic survival during early development [16]. Embryos homozygous for a null allele of *Elys* are resorbed at peri-implantation stages (e5.5–e7.5) and inner cell mass cells from cultured *Elys*−/−blastocysts undergo apoptosis soon after hatching from the zona pellucida. *Elys* is expressed throughout the developing mouse embryo and in a wide range of adult tissues [17], as is human ELYS (http://www.ncbi.nlm.nih.gov/UniGene/ESTProfileViewer.cgi?uglist=Hs.300887#Legend). Recently human and frog Elys proteins were shown to be orthologs of the gene encoding Mel-28, a protein required for nuclear integrity in C. elegans [18],[19]. Human and frog Elys proteins physically associate with the Nup107–160 nuclear pore protein complex [20],[21] and localize to kinetochores during mitosis, as has been described for other nuclear pore proteins (nucleoporins). RNAi mediated knockdown of ELYS protein inhibited nuclear pore complex (NPC) assembly, thus identifying ELYS as either a component of the NPC that directs its assembly, or a protein that organizes the NPC in the chromatin periphery. In addition to its association with the Nup107–160 nucleoporins, Elys interacts with the Mcm2-7 DNA helicase complex on chromatin derived from Xenopus egg extract [22]. This interaction was proposed to be a mechanism that allows cells to coordinate nuclear assembly with the requirement to shut down replication origin licensing prior to S-phase entry.

Here, we show that *flotte lotte* (*flo*), a previously described recessive zebrafish mutant with retinal, neural and digestive organ defects [23]–[26], arises from mutation of zebrafish *elys*. Strong maternal *elys* expression enables cells of early *flo* embryos to survive to larval stages. However, at later stages proliferative cells in tissues in which NPC assembly is disrupted, such as the retina, optic tectum and intestine, undergo cell cycle arrest and ultimately succumb to apoptotic cell death via p53-dependent and p53-independent mechanisms [27]. p53-independent apoptosis in the *flo* intestine is associated with activation of the Chk2 protein kinase [28], and initially, normal levels of the DNA damage marker γH2AX [29],[30]. This suggested that Elys may be needed to resolve replications errors that normally occur in highly proliferative organ progenitor cells. Consistent with this idea, we found that Chk2 activation was required for the survival of homozygous *flo* larvae, but not their heterozygous or homozygous wild type siblings. Homozygous *flo* larvae, but not their siblings, were also sensitized to DNA replication inhibitors, but not agents that induce DNA double strand breaks. Finally, we also found that loss of Elys reduces levels of chromatin bound Mcm2, but not Mcm3, Mcm4 or phospho-Mcm4 in the wild type or irradiated *flo* intestine. Together, these and other data support a role for Elys in DNA replication and the cellular response to replication stress, independent of its role in NPC assembly.

## Results

### The *flo* Mutation Disrupts Tissue Progenitor Cell Proliferation, Differentiation, and Survival

**Figure 1:**
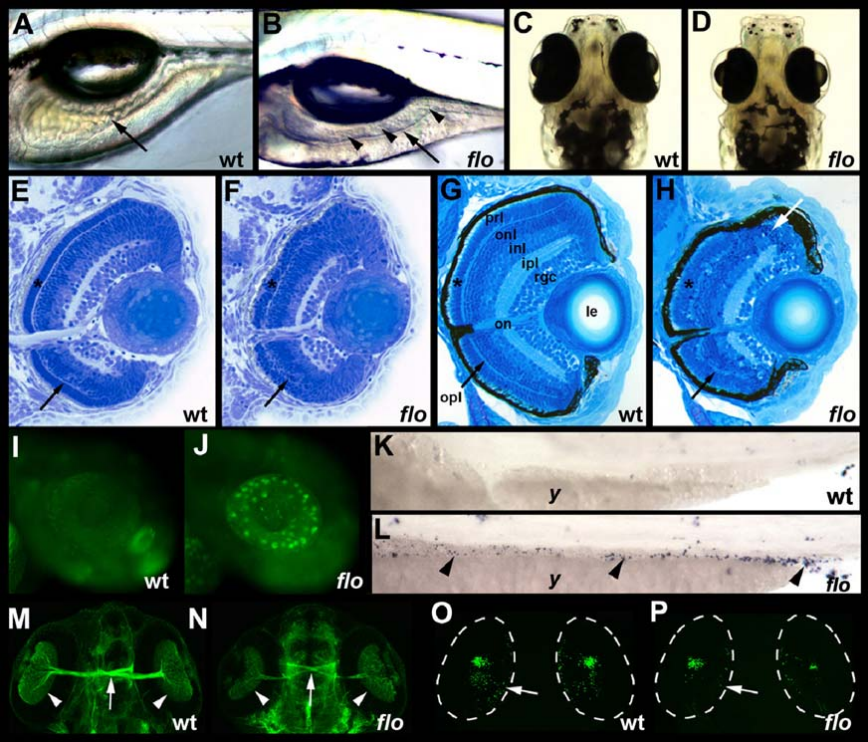
*flo* intestinal and retinal defects. (A,B) Lateral view of live 5 dpf wild type (wt) and *flo* larvae. The *flo* intestine lacks folds (arrow) and the lumen contains detached epithelial cells (arrowheads). (C,D) Dorsal view showing reduced size of the 5 dpf *flo* eye. (E,F) Histological cross section showing cells with condensed nuclei typical of apoptotic cells in the 60 hpf *flo* retina, and disorganization of the *flo* photoreceptor (*) and outer plexiform (arrow) layers. (G,H) Histological cross section showing marked disorganization of the 4 dpf *flo* retina and cells with condensed nuclei typical of apoptotic cells (white arrow). (I,J) Acridine orange staining showing apoptotic cells in the 48 hpf *flo* retina but not sibling wild types. (K,L) TUNEL staining showing apoptotic cells in the 75 hpf *flo* intestine (arrowheads) but not sibling wild types (anterior–left, posterior–right). (M,N) Dorsal view showing mild reduction in the number of *flo* retinal ganglion cells (arrowheads) and optic nerve diameter (arrow) identified with the Zn5 antibody. (O,P) Confocal projection of immunostained wt and *flo* larvae showing reduced rod cells in the *flo* retina including the large ventral cluster of cells and in the periphery of the mid retina (arrow). onl, outer nuclear layer; inl, inner nuclear layer; ipl, inner plexiform layer; rgc, retinal ganglion cell layer; on, optic nerve; le, lens; y, yolk.

Zebrafish *flotte lotte* (*flo^ti262c^*; hereafter *flo*) is a recessive lethal mutation that was identified as part of a large scale ENU mutagenesis screen [23]. Small eyes, optic tectum degeneration and digestive organ defects are prominent features of the *flo* mutant phenotype that are first evident in live fish on the fourth day post-fertilization (Figure 1A–1D). In previous work, the *flo* mutation was shown to disrupt differentiation and survival of 3 of the 4 principal intestinal epithelial cell lineages [24],[26], suggesting an effect on organ progenitor cells. Here, we show a comparable though less pronounced effect on retinal development (Figure 1E–1H). Histological analyses revealed that the cellular layers of the *flo* retinal epithelium were disorganized and contained numerous cells with condensed nuclei typical of apoptotic cells. Acridine orange and TUNEL staining revealed that apoptotic retinal (Figure 1I–1J) and intestinal epithelial cells (Figure 1K–1L) were already evident before *flo* mutants were morphologically distinguishable from wild type larvae. Immunohistochemical studies showed that differentiated retinal ganglion and photoreceptor cells were present in *flo* mutants, albeit at reduced levels compared with wild types, despite retinal disorganization and apoptosis (Figure 1M–1P). These studies suggested that early stages of retinal development proceed normally in *flo* mutants, most likely as a result of maternally derived mRNA and protein encoded by the affected gene (discussed below). To further assess the effect of the *flo* mutation on organ progenitors, we also measured cell proliferation in the intestinal and retinal epithelium. Both sets of data were consistent with G1 arrest of rapidly proliferating cells in these tissues (Tables S1 and S2). In summary, these descriptive analyses suggested a fundamental role for the *flo* gene in tissue progenitor cells. For this reason, a positional strategy was used to identify the responsible gene.

### *Flo* Encodes the Zebrafish *elys* Ortholog

**Figure 2:**
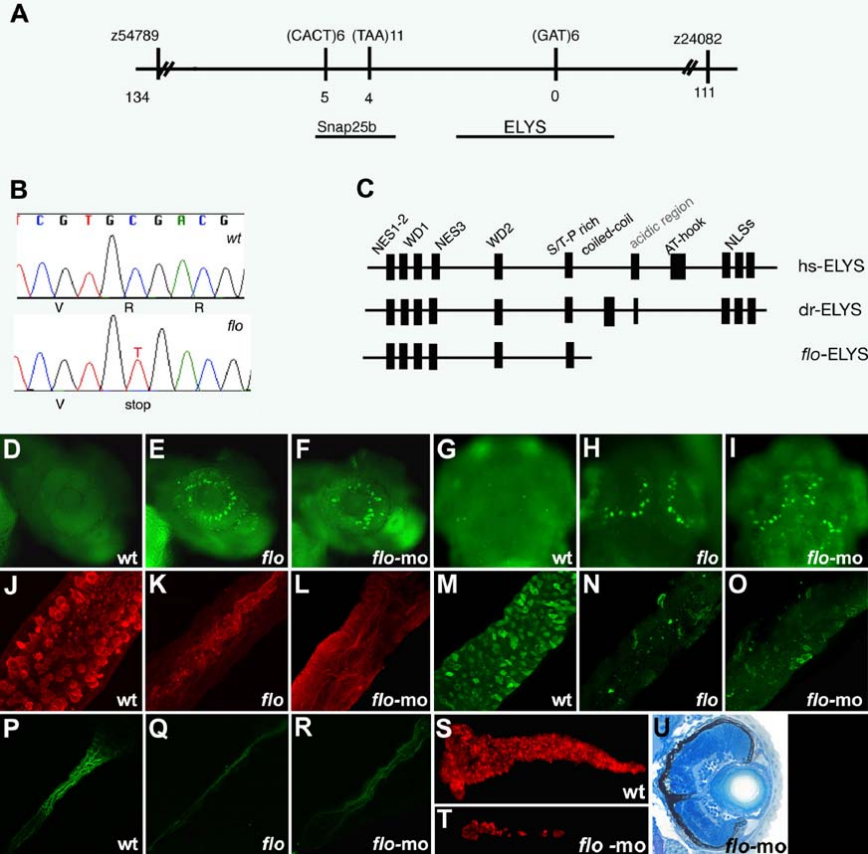
The *flo* locus encodes zebrafish *elys*. (A) Schematic representation of the genomic region surrounding the *flo* locus. The names of the polymorphic markers with the corresponding number of recombinants are listed. (B) DNA sequence analysis showing the cytosine to thymidine transition encoding the premature stop codon in the *elys^ti262c^* allele. (C) Schematic representation of the functional domains of the human (hs) and zebrafish (dr) Elys protein and the protein encoded by the *elys^ti262c^* allele (*flo-*ELYS). (D–I) Acridine orange staining showing apoptotic cells in the retina and growth plate of the optic tectum of 48 hpf *flo* (E,H) and *elys-*morpholino injected (F,I) larvae but not wt (D,G). (J–R) Confocal projections through the posterior intestine of 96 hpf larvae showing wheat-germ agglutinin positive goblet cells in the epithelium of the posterior intestine of wt (J) but not *flo* (K) or *elys-*morpholino injected (L) larvae; secretory cells in wt (M) but not *flo* (N) or *elys-*morpholino (O) injected larvae; enterocytes in wt (P) but not *flo* (Q) or *elys-*morpholino (R) injected larvae. (S–T) Carboxy-peptidase A positive cells are abundant in the 5 dpf wt (S) but not in *elys-*morpholino injected (T) exocrine pancreas. (U) Histological cross section through the retina of a 4 dpf *elys* morpholino injected larva showing retinal disorganization that is comparable to the 4 dpf *flo* retina (Figure 1H).

Bulk segregant analysis identified markers on zebrafish chromosome 17 that were linked to the *flo* locus (Figure 2 and Figure S1). Analyses of 2629 mutant larvae ultimately identified a zero recombinant marker within a predicted open reading frame that encoded a gene orthologous to mammalian *Elys* (also known as AT hook containing transcription factor 1; *ATHCF1*), a gene recently shown to be required for NPC assembly and nuclear integrity in worms [18]–[21]. *Elys* is expressed in a wide range of tissues and is essential for early mammalian development [16]. The longest open reading frame of zebrafish *elys* encodes a predicted protein that consists of 2527 amino acid residues. Computational analysis of the Elys protein revealed two WD-40 repeats and three nuclear export signals in the N-terminus plus a coiled-coil region (involved in protein-protein interactions) and several nuclear localization signals in the C-terminus (Figure 2C and Figure S2). The consensus sequence of the AT hook domain present in mammalian *Elys* was only partially conserved in the zebrafish ortholog. Sequence analyses of cDNA derived from *flo* larvae revealed a single base pair mutation encoding a cytidine to thymidine transition that generated a premature stop codon within the predicted zebrafish *elys* translation product (Figure 2B). This mutation is predicted to generate a truncated Elys protein that lacks 1209 amino acids containing the coiled-coil region and the nuclear localization signals. Protein truncation, coupled with reduced *elys* expression in *flo* larvae (Figure S3), most likely arising from termination codon associated mRNA decay [31], supports the idea that transcription of the *elys^ti262c^* allele generates only a small amount of active Elys protein.

**Figure 3:**
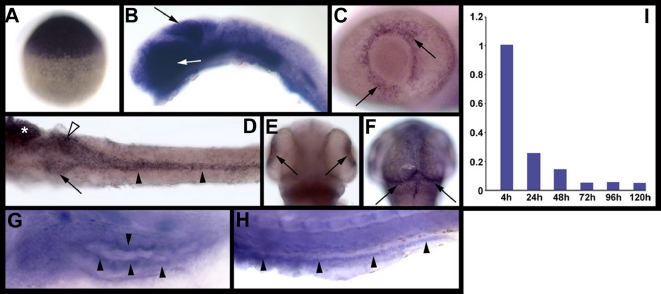
*elys* expression in developing zebrafish. Images (A–H) are whole mount RNA *in situ* hybridizations. (A) Maternal *elys* expression at 3 hpf. (B) Strong *elys* expression is evident in the midbrain (black arrow) and eye (white arrow) at 24 hpf. (C–F) Expression at 48 hpf in the retina (C,E), growth plate of the optic tectum (F), and digestive organs [(D): pancreas (arrow), anterior intestine (white arrowhead), posterior intestine (black arrowheads), liver (*)]. (G–H) Weak *elys* expression in the 5 dpf anterior (G) and posterior (H) intestine. (I) Graph showing *elys* expression in whole embryos (4 hpf–120 hpf) as determined via real-time quantitative PCR.

Antisense knockdown using morpholinos targeting either the *elys* 5′ UTR, translation initiation codon or the exon 30 splice acceptor (Figure S4) phenocopied the *flo* retinal (Figure 2D–2F and 2U) and tectal defects (Figure 2G–2I). Phenocopy was present in only a minority of embryos injected with single morpholinos (10%–12%), most likely because of the high levels of maternal *elys* mRNA relative to the levels of zygotically derived mRNA (Figure 3A). Combined injection of the 5′-UTR and exon 30 splice donor morpholinos led to phenocopy in 30% to 50% of injected embryos (n = >300 injected embryos). All *elys-*morpholino injected larvae with retinal and tectal apoptosis had minimal exocrine pancreas tissue (Figure 2S–2T) as do *flo* larvae [25]. Similarly, like *flo* mutants [4], all of the affected morpholino injected larvae lacked intestinal goblet cells (Figure 2J–2L) and had a dramatic reduction in non-enteroendocrine secretory cells and enterocytes (Figure 2M–2R) as revealed by previously described monoclonal antibodies [32]. Apoptosis, which is a prominent feature of the *flo* intestinal phenotype (discussed below), was not evident in the intestine of the *elys-*morpholino injected larvae despite clear evidence of NPC disruption. Although partial intestinal phenocopy by the Elys knockdown was not unexpected because morpholino knockdowns are often transient, these data raise the possibility that apoptosis caused by loss of Elys function may occur independently of altered NPC assembly seen in *flo* mutants.

### Developmental Pattern of *elys* Expression

The pattern of *elys* expression in zebrafish embryos and larvae is consistent with nearly all aspects of the *flo* phenotype (Figure 3). As mentioned previously, high levels of maternal RNA encoding *elys* are present in newly fertilized zebrafish embryos (Figure 3A). This could account for the absence of an early zygotic phenotype that was reported in *Elys* knockout mice. Early zebrafish embryos (24 hours post-fertilization; 24 hpf) show strong *elys* expression in the brain (Figure 3B), whereas at 48 hpf expression was largely restricted to the retina (Figure 3C), the growth plate of the optic tectum (Figure 3E–3F) [33], and the digestive organs (Figure 3D), tissues that are all highly proliferative at this stage [24],[34],[35]. *Elys* expression in the zebrafish digestive organs persists through 5 days post-fertilization (dpf), when cell proliferation in these organs is low, but at a greatly reduced level compared to 48 hpf, when the rate of cell proliferation is high (Figure 3G–3H) [24]. This dynamic expression pattern fits with the recently described role of *elys* in NPC assembly, since NPC turnover is low in non-proliferating cells [36].

### Altered Nucleoporin Distribution in the *flo* Retina and Intestine

**Figure 4:**
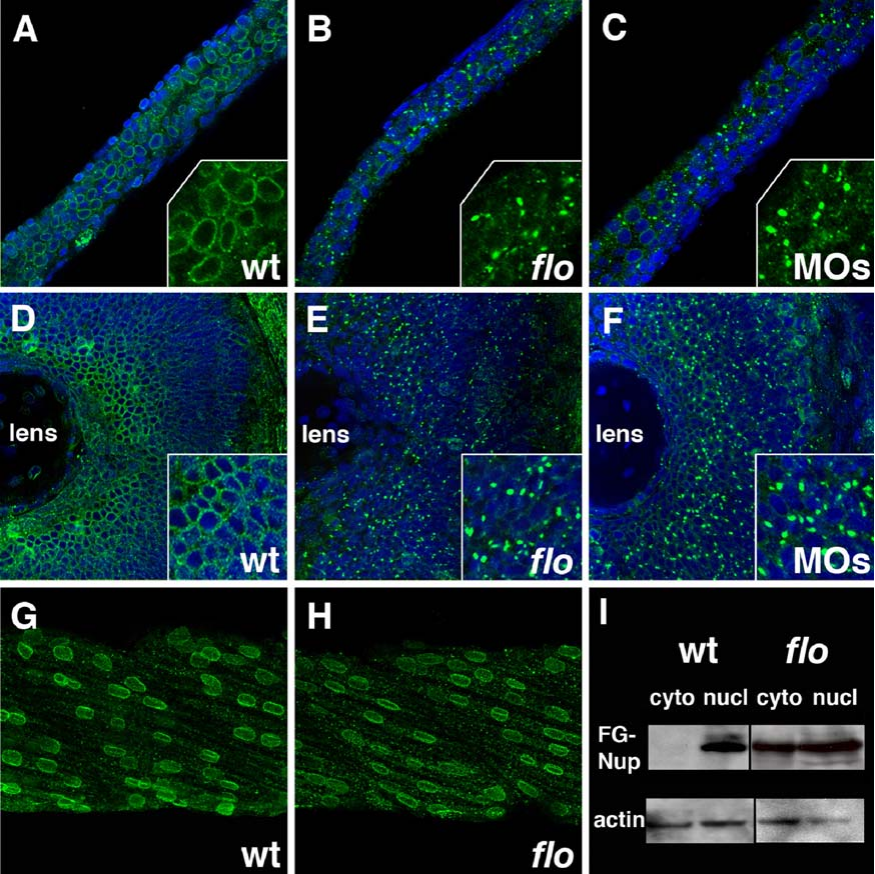
Nuclear pore disruption in *flo* mutants. (A–C) Confocal projections through the posterior intestine of 75 hpf wild type (A), *flo* (B), and *elys* morpholino injected larvae (C), following anti-FG nucleoporin immunostainings with mAb414 (green; DAPI–blue). There is a dramatic reduction of nuclear pores in the *flo* and morpholino injected larvae. Inset shows higher magnification of localized regions of the DAPI-stained image. (D–F) Identical findings are evident in the retina of these larvae. Note apparent cytoplasmic accumulation of the immunoreactive FG-nucleoporins in *flo* and morpholino injected larvae. (G,H) Normal nuclear distribution of FG nucleoporins in wild type and *flo* skeletal muscle. (I) Western blot showing levels of FG nucleoporin proteins relative to beta-actin in nuclear (nucl) and cytoplasmic (cyto) extracts derived from the intestine of 75 hpf *flo* and sibling wild type larvae.

**Figure 5:**
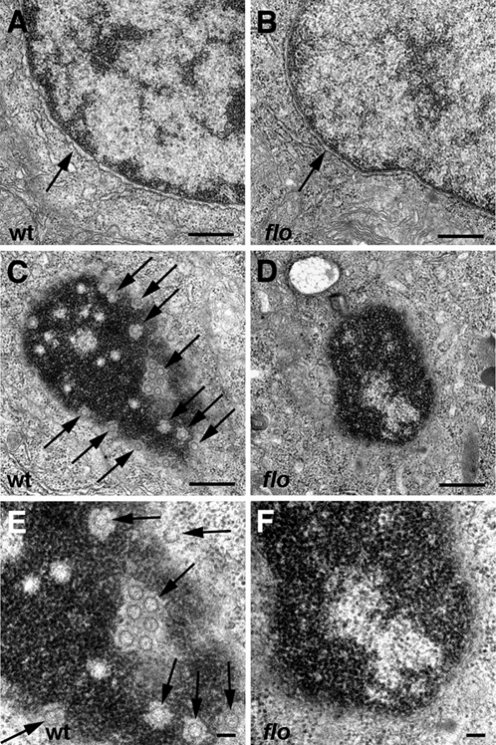
Nuclear ultrastructure in *flo* mutants. Transmission electron micrographs of nuclei from representative 5 dpf wild type and *flo* intestinal epithelial cells. (A,B) Intact nuclear envelope in wild type (A) and *flo* (B). (C–F) Tangential sections through the nuclear envelope showing abundant nuclear pores (arrows) in the wild type larva (C,E) but few if any well defined pores in the *flo* larva (D,F). (E) and (F) are higher magnification views of (C) and (D), respectively.

NPC disassembly caused by *ELYS* knockdown in HeLa cells leads to redistribution of nucleoporins from the nuclear envelope to the cytoplasm [20]–[21]. Immunohistochemical analyses of zebrafish nucleoporins using a monoclonal antibody that recognizes nucleoporins with a highly conserved FG domain [37] showed typical nuclear staining in all wild type zebrafish tissues. By contrast, a dramatic reduction of nuclear FG-nucleoporins was evident in *flo* intestinal and retinal epithelial cells (Figure 4A–4F). A normal FG-nucleoporin immunostaining pattern was clearly evident in *flo* skeletal muscle (Figure 4G–4H), pronephric duct epithelia and other non-proliferative tissues (data not shown). The *flo* retinal NPC defects were evident as early as 36 hpf, and the intestinal NPC defects could be seen at 48 hpf (Figure S5). FG-nucleoporin immunostainings from *flo* and *elys* morpholino injected larvae were nearly identical and closely resembled those of ELYS-deficient HeLa and U2OS cells [20]–[21] thus suggesting partial cytoplasmic redistribution of these nucleoporins. Western analyses of nuclear and cytoplasmic fractions of *flo* intestinal proteins confirmed these findings (Figure 4I). Similarly, ultrastructural analyses showed a marked reduction in the number of identifiable NPCs in the *flo* intestinal epithelial cells (Figure 5) and also showed that the *flo* mutation had no effect on nuclear envelope formation or stability, as was reported in ELYS-deficient mammalian cells [21]. Together, these data confirm a role for zebrafish Elys in NPC assembly.

### The *flo* Mutation Causes Both p53-Dependent and p53-Independent Apoptosis and Cell Cycle Arrest

**Figure 6:**
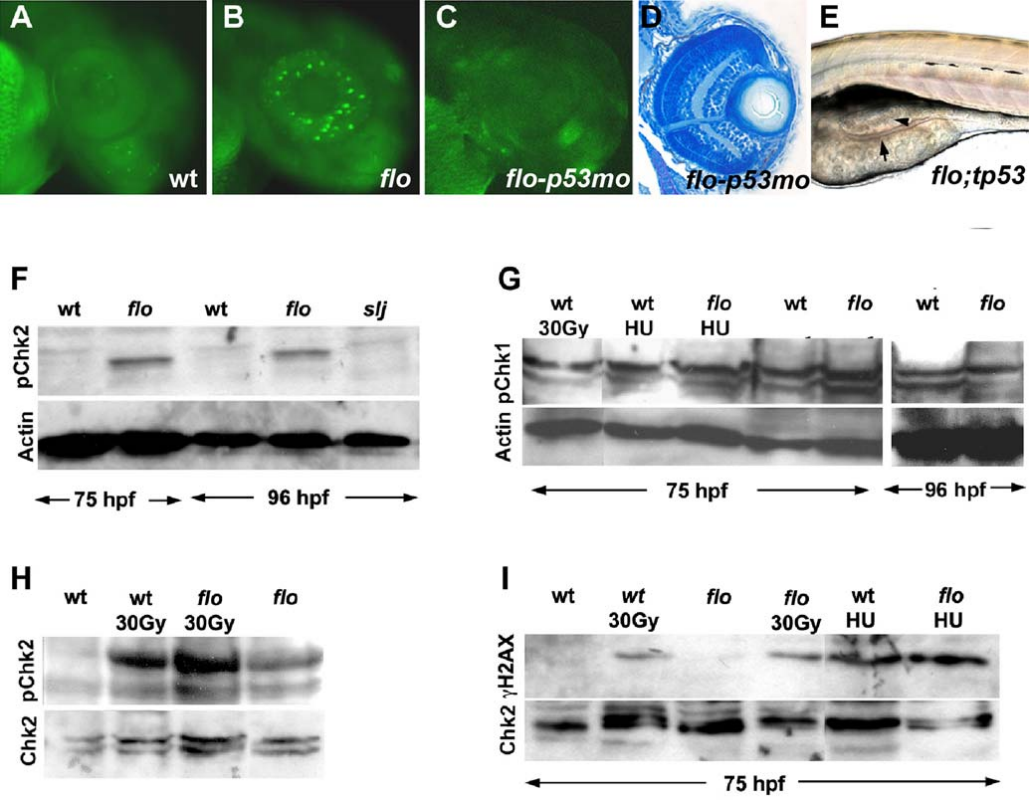
The *flo* mutation activates the DNA damage response. (A–C) Acridine orange staining showing apoptosis in the 50 hpf *flo* retina that is rescued by the *tp53* morpholino (mo) knockdown. (D) Histological cross section showing rescue of *flo* retinal architecture defects by tp53 knockdown [compare (F) with Figure 1G and 1H]. (E) Intestinal defects persist in *flo/tp53* double mutants. Arrow, thin intestinal wall; Arrowhead, apoptotic cells in the intestinal lumen. (F) Western blot showing elevated levels of phospho-Chk2 (Serine 33) in the intestine of *flo* larvae, compared with sibling wild type larvae, but not *slim jim* larvae (I). (G) Western blot showing comparable levels of phospho-Chk1 (Ser 345) in *flo* and sibling wild type larvae, before and after γ-radiation (30 Gy) and treatment with hydroxyurea (HU). (H) Western blot showing enhanced phospho-Chk2 activation in the intestine of *flo* and wild type larvae following γ-radiation (30 Gy). (I) γH2AX is not detected in the *flo* or wild type intestine (75 hpf), but is detected at this stage following γ-irradiation (30 Gy) or hydroxyurea treatment (HU).

To gain a better understanding of how NPC disruption caused apoptosis and cell cycle arrest in *flo* intestine and retina, we assayed expression of *tp53* and *p21* in 50 hpf and 75 hpf *flo* mutants via real-time quantitative PCR. These experiments showed increased expression of *tp53*, *p21* and also *mdm2*, a negative regulator of p53 whose expression is induced in response to p53 activation (Figure S6). To further assess the role of *tp53* in *flo* mutants, we injected antisense morpholinos known to target zebrafish *tp53* mRNA translation [27],[38],[39] into newly fertilized embryos derived from matings of heterozygous *flo/+* fish. The *tp53* knockdowns restored normal *p21* and *tp53* expression in *flo* larvae. We also generated larvae that were homozygous for both the *flo* mutation and a previously described *tp53* mutation that inhibits p53-dependent radiation induced apoptosis [40]. The homozygous *tp53* mutation as well as the *tp53* knockdowns rescued retinal apoptosis in 50 hpf *flo* mutants (n = 25 *flo/tp53* double mutants analyzed and >200 *tp53* morpholino injected larvae analyzed; Figure 6A–6C). Retinal size and architecture was restored or greatly improved by the *tp53* knockdown and mutation in most *flo* larvae (n>50, 5 dpf larvae examined; Figure 6D), but neither rescued intestinal morphology, differentiation or apoptosis (Figure 6E; n = 25 *flo/tp53* double mutants analyzed at 4 dpf). These data show that disruption of NPCs activates the DNA damage response in a tissue-specific manner.

### Chk2 Is Activated in *flo* Intestine and May Be Responsible for p53-Independent Cell Cycle Arrest and Apoptosis

Levels of activated Chk1 and Chk2 protein kinases were assayed in the *flo* intestine to determine whether they could account for *tp53*-independent apoptosis. Mammalian Chk2 is activated by the ATM kinase in response to double strand DNA breaks and other types of DNA damage [28]. Phospho-Chk2 has been reported to induce cell cycle arrest and apoptosis via both p53-dependent and p53-independent signaling pathways [28],[41]. Antibodies to phosphorylated and non-phosphorylated mammalian Chk2 recognized 55 kD zebrafish proteins in nuclear extracts from wild type and *flo* intestine, and morpholino knockdown confirmed the specificity of the anti phospho-Chk2 antibody (Figure S6). Levels of phospho-Chk2 (Figure 6F), not phospho-Chk1 (Figure 6G; lanes 4, 5) were elevated in the *flo* intestine, whereas phospho-Chk2 levels were normal or only slightly elevated in the *flo* retina (Figure 6). To get a better understanding of the cause of Chk2 activation in *flo* intestine, we assayed phospho-Chk1 and phospho-Chk2 levels in response to two types of stimuli, γ-irradiation induced double strand DNA breaks and replication arrest induced by the nucleotide synthesis inhibitor hydroxyurea. Unlike mammalian cells, we found that the levels of phospho-Chk1 in wild type intestine were not increased upon treatment with hydroxyurea or γ-radiation, whereas phospho-Chk2 levels were increased in response to both. (Figure 6G–6H and data not shown). This suggests that intestinal progenitor cells in zebrafish larvae rely on Chk2 rather than Chk1 to activate checkpoints in response to both DNA damage and replication inhibitors. We speculate this may be because endogenous replication stress already maximally activates Chk1 in these rapidly proliferating cells (a 1 hr incubation with BrdU labels >25% of intestinal epithelial cells; [24] and Table S1).

### Chk2 Activation in the *flo* Intestine Is Essential for Survival

Checkpoint proteins are typically activated in the setting of DNA damage. For this reason, we measured levels of γH2AX, a phosphorylated form of the Histone 2A variant H2AX that accumulates in cells in response to double strand breaks and DNA damage induced by ionizing radiation and other agents [29],[30]. Western and immunohistochemical analyses showed that γH2AX was either not detectable or present at very low levels in the 75 hpf wild type and *flo* intestine (Figure 6I, lanes 1, 3; and data not shown). γH2AX and increased levels of phospho-Chk2 were both detectable, however, in the intestine of 75 hpf wild type and *flo* larvae 4 hours after γ-irradiation and treatment with hydroxyurea (Figure 6H–6I), thus confirming an intact damage response in the absence of normal Elys function. Together, these data argue that Chk2 activation in the intestine of early *flo* larvae occurs in the setting of low level DNA damage such as may be expected to occur in the setting of replication stress.

To better understand why Chk2 is activated in the *flo* intestine, we inhibited its function using a commercially available pharmacological inhibitor. Pretreatment of wild type zebrafish larvae with this inhibitor prevented phosphorylation of Chk2 at a conserved serine residue normally phosphorylated by ATM (Figure S6). An identical phosphorylation response has also been reported to occur with mammalian Chk2 inhibition [42], thus supporting the activity of this inhibitor against zebrafish Chk2. Twelve hour treatment with this inhibitor was well tolerated by 4 dpf wild type (+/+) and heterozygous *flo* larvae, although in two independent experiments the treatment induced apoptosis in the intestinal epithelium of ∼50% of larvae analyzed (n = 285 larvae). By contrast, concomitant treatment was lethal by 24 hours post-treatment for all sibling homozygous *flo* larvae analyzed (n = 86 larvae), most likely as a result of widespread intestinal injury. Unlike *flo*, Chk2 inhibition was well tolerated by another zebrafish mutant, *slim jim* (0% lethality of 80 mutant larvae) in which intestinal epithelial cell proliferation is reduced to a level comparable to *flo* as a result of altered RNA Polymerase III activity [43]. This lack of effect of Chk2 inhibition in *slim jim* mutants is expected because in contrast to *flo*, phospho-Chk2 levels are not elevated by this mutation (Figure 6F). These data argue that checkpoint activation in *flo* larvae occurs in response to physiological stimuli.

To further interrogate the ATM-Chk2 pathway, *flo* and sibling wild type larvae were treated with a commercially available ATM inhibitor. Overnight treatment of 82 hpf larvae had a profound effect on the development and survival of *flo* mutants and their siblings; nearly all larvae died or had pronounced cardiac and neural defects (data not shown). Although a modestly more pronounced effect was evident in the *flo* larvae, the difference in sensitivity to ATM inhibition was minor in comparison to the differential effects of Chk2 inhibition. Treatments with caffeine, an inhibitor of the mammalian ATR-Chk1 signaling pathway was tolerated by *flo* and sibling larvae, but caused significant developmental delays in both groups (data not shown).

### Sensitivity of *flo* Larvae to Selected Replication Inhibitors Points to a Role for Elys in Resolving DNA Replication Errors

Chk2 dependence of *flo* larvae that have only low γH2AX levels suggested that Elys could be required to maintain replication forks in highly proliferative tissue progenitor cells. To test this hypothesis, we treated wild type and *flo* larvae with hydroxyurea, a DNA replication inhibitor that can activate Chk2 (as well as Chk1) and H2AX in mammalian cells [44] and zebrafish larvae (data not shown). Homozygous *flo* larvae were sensitized to hydroxyurea treatment. Overnight or 6 hour exposure to hydroxyurea doses (250 mM) was lethal for all homozygous *flo* larvae analyzed (n = 86 larvae) but was well tolerated by all sibling wild type and heterozygous *flo* larvae analyzed (n = 302 larvae), as well as *slim jim* larvae (n = 60 mutant larvae). Survival of *flo* mutants, but not their wild type siblings or *slim jim* mutants, was also reduced following treatment with the DNA cross-linker cisplatin (10 uM; 12 hour treatment) as well as UV-irradiation, another cause of replication stress, but not the DNA topoisomerase 1 inhibitor camptothecin, even at doses that caused widespread intestinal apoptosis and wild type larval death (5–20 uM; 12 hrs), or γ-irradiation (30 Gy). Together, these data suggest that the Elys protein may be required to prevent replication fork collapse in response to specific types of replication inhibitors. The effects of Chk2 inhibition and replication inhibitors on *flo* mutants are listed in Table 1.

**Table 1:** Effect of DNA replication inhibitors on wt and *flo* larvae.

Inhibitor	Concentration Duration	WT Treated/Survived	*Flo* Treated/Survived
Hydroxyurea	250 mM/12 hrs	302/302	86/0
Chk2 Inhibitor	10 uM/12 hrs	285/285	86/0
UV Irradiation	10 minutes	324/264	124/0
Cisplatin	10 uM/12 hrs	384/365	162/0
Camptothecin	10 uM/12 hrs	212/0	114/0

Wild type (WT) and *flo* larvae treated beginning at 84 hpf for the duration listed. Treatment effect scored 12–24 hrs post-treatment. Lethality defined as severely reduced or absent cardiac contraction and blood circulation, or pronounced neural degeneration or morphological deformity.

### Loss of Elys Function Reduces Chromatin Bound Mcm2, a Component of the DNA Replication Helicase

**Figure 7:**
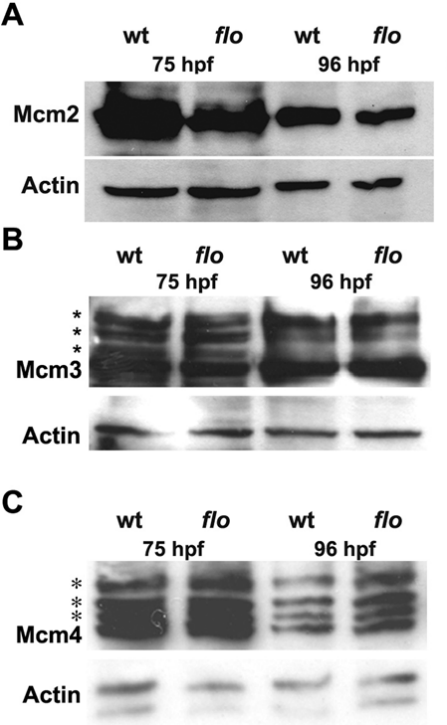
Reduced chromatin bound Mcm2 in the *flo* intestine. (A) Western analysis showing reduced levels of chromatin bound Mcm2 in 75 hpf and 96 hpf *flo* intestine compared with wild type siblings. By contrast, levels of chromatin bound Mcm3 (B) and Mcm4 (C) in the *flo* intestine are comparable to wild type. Multiple bands (*) corresponding to phospho-Mcm3 and phospho-Mcm4 are recognized by the antibodies directed against the native proteins in wt and *flo* samples.

Recently, the Mcm2-7 complex, a central component of the DNA replication helicase, was shown to bind Elys and promote its loading onto chromatin at the M-G1 transition of the cell cycle [22]. Reconstitution of the NPC complex was postulated to promote the nuclear import of Geminin, a Cdt1 inhibitor, at the onset of S-phase, thus preventing reloading of Mcm2-7 onto chromatin and DNA re-replication. These data were the first to link replication licensing with NPC assembly. Despite the absence of nuclear pores in *flo* retinal and intestinal epithelial cells, we found no evidence of promiscuous firing of licensed replication origins in these highly replicative cells, as arrest in G1 rather than S phase was evident in both tissues (Table S2). However, biochemical analyses did reveal an effect of Elys deficiency on Mcm-chromatin interactions. These showed significantly reduced levels of chromatin bound Mcm2 were present in the *flo* intestine at 75 hpf and 96 hpf (Figure 7A and Figure S6). By contrast, levels of Mcm3 and Mcm4 in *flo* mutants were either normal or minimally reduced at both time points (Figure 7B–7C), and Mcm2 levels were only minimally reduced in intestinal cells of *slim jim* mutants, which have reduced proliferation and undergo cell death (Figure S6). Levels of phospho-Mcm4, which are elevated in response to replication stress [45],[46] were also normal in cells derived from irradiated *flo* intestines (Figure S6). Together, these data link Elys deficiency with reduced chromatin loading or maintenance of chromatin binding of selected Mcm2-7 complex proteins.

## Discussion

### Evolutionarily Conserved Role for Elys in Vertebrate NPC Assembly

Nucleocytoplasmic transport enables macromolecules such as messenger and non-coding RNAs, transcription factors, and other proteins to traverse the nuclear envelope. The NPC, which consists of over thirty proteins (nucleoporins), plays an essential role in mediating and regulating this process [36],[47]. Genetic analyses in fungi, worms, flies and mammals have shown that many nuclear pore proteins are required during early development. This developmental role is thought to largely be a function of the pore's effect on transport. Recent studies however, have identified nucleoporin functions not associated with transport that may influence other cellular processes, such as the function or assembly of spindles and kinetochores, post-translational protein modification, and chromatin organization, which can affect gene transcription and DNA repair [36],[47],[48]. The disruption of these processes may play a contributory role to the NPC deficient phenotype.

Here, we report the phenotype arising from mutation of zebrafish *elys*, a gene recently shown to be required for NPC assembly in human cells that was previously known to be essential to early mouse development [16]. We speculate that *flo* embryos survive early development because of the high level of maternal *elys* expression. As a result, the effects of the loss of Elys protein function during the later developmental stages, such as organogenesis, can be assayed. The principal findings of our study regarding the role of Elys protein in NPC assembly are in agreement with the reported role of Elys in human cells and in vitro assays of NPC assembly using siRNA knockdown and competitive binding assays [20]–[22]. Using ultrastructural and immunohistochemical analyses, we show that mutation or knockdown of Elys disrupts nuclear pore assembly in rapidly proliferating cells. By contrast, NPC assembly and maintenance appear normal in quiescent cells that stop cycling before stores of maternally derived Elys protein are depleted. These findings conform with studies performed in cultured cells which showed that NPCs are stable until their disassembly during mitosis [49].

The *elys^flo^* point mutation introduces a premature termination codon into the Elys cDNA, upstream of the coiled-coil domain, the AT hook region and 3′ nuclear localization signals. Absent an antibody that recognizes the zebrafish Elys protein, we cannot determine if such a protein is actually translated from reduced levels of mRNA we can detect in *flo* mutants. Furthermore, whether such a truncated protein might retain activity was not addressed in this study. Based on comparison with the NPC phenotype arising form Elys knockdown in cultured cell lines, the *elys^flo^* allele appears to be either a severely hypomorphic or null allele. In the future, further analysis of this or other *elys* alleles may be used to dissect functional domains in the Elys protein that might mediate pore and non-pore related functions (discussed below).

### Chk2 Is Activated in the *flo* Intestine in Response to DNA Replication Stress

Apoptosis is a prominent feature of the *flo* mutant phenotype. We focused on understanding the cause of cell death in the *flo* intestinal epithelium because of our prior work using the zebrafish to study digestive organ development. Our experiments showed that apoptosis in the *flo* intestine was not dependent on p53, whereas it was in the retina and tectum. Instead Elys deficiency activated Chk2, a checkpoint protein that can induce cell cycle arrest and apoptosis independently of p53. Checkpoint activation is an important component of the cellular response to replication stress, because it allows stabilization of stalled replication forks, thereby averting replication fork collapse and activation of apoptotic programs [50]–[51]. In mammalian cells replication stress activates the Chk1 checkpoint protein whereas double strand breaks are considered to be the primary stimulus for Chk2 activation [28]. For this reason, Chk2 activation in *flo* mutants was unexpected. γ-irradiation of zebrafish larvae induced Chk2 phosphorylation, thus showing an evolutionarily conserved role for this arm of the DNA damage response pathway in zebrafish larvae. Chk2 was also activated by replication stress induced by hydroxyurea, whereas Chk1 phosphorylation was unchanged from its baseline level of activation with either treatment. From this, we conclude that hydroxyurea induced replication stress activates Chk2 in the zebrafish intestine because Chk1 is already maximally activated by endogenous replication errors that occur in these highly proliferative progenitor cells [24].

Although we initially postulated a number of mechanisms to explain how mutation of Elys activated the DNA damage response, several lines of evidence support a model in which Elys protein is required for the repair of replication errors that occur in rapidly dividing cells. First, Chk2 inhibition was lethal to *flo* larvae even though we found evidence of only low levels of DNA damage in the mutant larvae at this stage (normal γH2AX levels). This argued that checkpoints were activated in response to physiological stimuli; i.e. - replication stress. Second, survival of *flo* mutants was reduced following treatment with doses of replication inhibitors, such as hydroxyurea, UV-irradiation and cisplatin, that had only minor effects on wild type siblings, or another mutant, *slim jim*, in which intestinal progenitor cells proliferation is also reduced. These data show that Elys deficient cells cannot tolerate additional genotoxic insults induced by stalled DNA replication forks, as reported for cells carrying mutations of DNA repair genes [44]. In contrast to their sensitivity to replication inhibitors, treatment of *flo* mutants with γ-irradiation or the topoisomerase inhibitor camptothecin did not compromise survival compared with wild type siblings. Non-sensitivity to these agents, which induce DNA double strand breaks [52], argues that *flo* larvae retain the ability to activate repair pathways involving homologous recombination or non-homologous endjoining, whereas mechanisms to maintain replication forks in the setting of nucleotide deprivation (hydroxyurea) or intrastrand DNA crosslinks (UV, cisplatin) are compromised by Elys deficiency. Collectively, these data argue that sensitivity of Elys deficient cells to hydroxyurea and other agents is not a non-specific effect incurred by cells that lack nuclear pores.

### Reduced Mcm2 in *flo* Intestine May Impair Licensing of Dormant Replication Origins

Levels of Mcm2-7 complex proteins in eukaryotic cells far exceed the number of active replication origins [53]. In both worms and human cells, excess Mcm2-7 complexes license dormant origins for firing when nearby replication forks are irreversibly stalled, thus preserving DNA replication and cell viability in the setting of replication stress [53],[54]. Mcm proteins also promote replication fork stability in fission yeast [46]. We found that levels of Mcm2, but not Mcm3 or Mcm4, were significantly lower in chromatin preps derived from *flo* intestinal cells compared with both wild type siblings and *slim jim* mutants that had a comparable reduction of intestinal cell proliferation. These data argue that reduced Mcm2 levels are caused by Elys deficiency, rather than reduced proliferation, as previously reported [55]. Reduced Mcm2 levels associated with Elys deficiency could arise from an effect on Mcm2-chromatin loading, or maintenance of chromatin bound Mcm2. We favor an effect on the maintenance of Mcm2-chromatin interaction since a peptide inhibitor of Elys chromatin binding did not affect binding of Mcm2 in Xenopus egg extracts [22].

Mcm protein levels vary with the cell cycle, and we initially considered this as a possible explanation for reduced Mcm2 levels in *flo*. However, G1 arrest induced by Elys deficiency would be expected to increase rather than decrease levels of chromatin bound Mcm2-7 complex [22]. Mcm levels have been reported to fall when cells enter replicative senescence [56],[57]. However, senescence is associated with a reduction in the levels of all Mcm proteins [57], whereas we found that only levels of Mcm2 were significantly reduced in *flo* mutants. Also arguing against this mechanism, we found that Mcm levels were not reduced in *slim jim* mutants that had a comparable reduction in the percentage of cycling cells that had arrested in G1 [43]. Finally, reduced Mcm chromatin loading is predicted to occur upon DNA-damage induced degradation of the licensing factor Cdt1. However, this too is expected to reduce binding of all Mcm proteins.

We speculate that reduced levels of chromatin bound Mcm2 prevents firing of dormant replication origins that sustain DNA replication stalled by endogenous replication errors, or stress arising from exogenous agents, such as hydroxyurea. When this compensatory mechanism is impaired, stalled replication forks are more likely to collapse, leading to cell death. Consistent with this idea, mice homozygous for a non-lethal hypomorphic *Mcm2* allele have reduced numbers of proliferating progenitor cells in the subventricular zone of the brain [58]. As the surviving progenitor cells cycle normally, their reduced census argues that surviving progenitor cells were able to overcome endogenous replication stress using an alternative salvage mechanism. Interestingly, a comparable effect on progenitor cell survival was noted in this study in the intestine of the Mcm2 mutant mice. Activation of dormant origins of human cells exposed to hydroxyurea is also disrupted by partial Mcm5 knockdown [54]. Unlike zebrafish Elys deficiency, partial Mcm5 knockdown (75% reduction) reduced levels of all other Mcm proteins analyzed, including Mcm2 (50% reduction), which may explain why survival of these cells was reduced by treatment with hydroxyurea and camptothecin whereas *flo* mutants were only affected by hydroxyurea.

Mcm2-7 proteins form a hexameric complex *in vivo*, but only the Mcm4,6,7 subcomplex, and not the Mcm2,3,5 subcomplex has helicase activity *in vitro*. Recent work suggests that the Mcm2,3,5 subcomplex may regulate the association of hexameric Mcm2-7 complex with chromatin, and its helicase activity *in vitro*, through the phosphorylation of Mcm2 and or Mcm5 by the CDC7/DBF4 kinase [59]. Thus, it is conceivable that reduced Mcm2 in Elys-deficient cells impairs helicase activation at dormant origins when nearby replications forks are stalled by endogenous replication errors. As noted previously, since a peptide derived from the Elys-chromatin binding domain did not impair chromatin loading of Mcm2-7 [22], we speculate that maintenance of chromatin bound Mcm2 involves interaction with another region of the Elys protein, other members of the NPC or an unrelated mechanism.

### A Potential Role for Elys in DNA Replication, Independent of Its Role in NPC Assembly

We recognize that the lack of nuclear pores could play a significant role in the response of *flo* tissue progenitor cells to both endogenous and exogenous replication stress. Reduced nuclear export stabilizes p53 in response to genotoxic stress [60],[61] and this could be enhanced in Elys-deficient cells, thus contributing to p53-dependent apoptosis in the retinal and optic tectum. Similarly, mutation of the ALADIN nucleoporin gene has been reported to sensitize cells to pharmacologically induced single strand breaks through a transport mechanism [62]. It is also conceivable that inhibition of NPC assembly affects DNA repair by altering chromatin configuration and secondarily, gene expression, as reported in yeast carrying non-lethal nucleoporin mutation [63]. Indeed, chromatin condensation was evident in Xenopus nuclear extracts depleted of Elys [21]. Electron micrographs of *flo* intestinal and retinal cells showed no evidence of gross chromatin defects, however, this does not exclude the possibility that zebrafish NPC disruption alters chromatin organization, such as the location of heterochromatin – euchromatin boundaries, which can epigenetically alter gene transcription.

Despite these considerations, two experiments from this study argue that a lack of nuclear pores may not be sufficient to affect a cell's ability to respond to replication stress. First, p53-mutation rescued apoptosis in the *flo* retinal epithelium even though the NPC defect persisted in all of the double mutant cells we analyzed. Second, morpholino knockdown of *elys* fully phenocopied the NPC and differentiation defects present in *flo* mutants, but did not cause apoptosis. Thus, in both the eye and intestine, the nuclear pore defect associated with Elys deficiency could be dissociated from apoptosis. These findings are interesting in light of genetic studies in yeast and other fungi that first indicated a role for nucleoporins in DNA repair: non-lethal nucleoporin mutations can cause either sensitization to DNA damaging agents [64]–[66] or synthetic lethality when combined with mutations in DNA repair genes [67]. Given binding of Elys to Mcm proteins [22], collectively these data suggest a role for Elys, and conceivably other nucleoporins, in chromatin interactions that are important for DNA replication in highly proliferative cells.

Although it may be difficult to fully exclude a role for the NPC in the *flo* phenotype, one way to further address the role of Elys in DNA replication would be to generate *elys* alleles that preferentially sensitize progenitor cells to replication stress, rather than NPC assembly. This may identify regions of the Elys protein that are required for Mcm-chromatin interactions, but not NPC assembly. We plan to pursue this type of genetic screen and other experiments that can help characterize other interesting features of the *flo* mutant phenotype, such as the tissue specificity of p53-dependent apoptosis and Chk2 activation, in future studies.

## Materials and Methods

### Zebrafish Stocks and Treatments

Wild type, heterozygous *flo* (*flo^ti262c^*, hereafter *flo*) and *p53* (*tp53^zdf1^*) adult fish were maintained as described [24]. Wild type and *flo* larvae were treated with hydroxyurea (Sigma Aldrich) and the Chk2 inhibitor (Sigma Aldrich) at the indicated concentrations for 4 hours and overnight respectively. To identify apoptotic cells, larvae were soaked in acridine orange (5 ug/ml) for 30 minutes, rinsed in embryo media and examined under a fluorescent dissecting microscope (Olympus MVX 10).

### Genetic Mapping and Positional Cloning of the *flo* Locus

Chromosomal localization of the *flo* locus was performed using bulk segregant analysis as previously described [68]. Details of high resolution genetic mapping of the *flo* locus are presented as Text S1.

### Genotyping of *flo* Embryos and Larvae

Homozygous *flo* larvae were identified via PCR amplification of genomic DNA using primers designed to create Afl III and Hph I recognition sites at the site of the *flo* mutation using dCAPS Finder 2.0 (http://helix.wustl.edu/dcaps/dcaps.html). Primer sequences are supplied in Text S1.

### RT-PCR and Quantitative RT-PCR

RNA recovery and RT-PCR was performed as described [69]. RNA from fifty embryos or dissected tissue samples was used in each experiment. Prior to 4 dpf, *flo* larvae were identified by molecular genotyping or retinal acridine orange staining. Primers used for RT-PCR reactions are listed in Text S1.

### Morpholino Antisense Knockdown

Morpholino antisense oligonucleotides (Genetools; Open Biosystems) were injected into one-cell stage wild type embryos as described [68]. In some cases, the morpholinos were also injected into the yolk of 24 or 48 hours post-fertilization (hpf) embryos. The sequences of *elys* and *tp53* morpholinos are supplied in Text S1.

### Histological Analyses

Histological and immunohistochemical analyses were performed as described [24],[25]. Enterocytes and secretory cells were identified using 4E8 and 2F11 monoclonal antibodies [32]. Zn-5 antibody was obtained from the Zebrafish International Resource Center. The 1D1 anti-rhodopsin antibody (photoreceptor cells) was a gift of James Fadool. Monoclonal antibody mAb414 (Covance, Berkeley, CA) was used to recognize FG-nucleoporins.

### Western Analyses

Nuclear protein was recovered from the intestine or eye of fifty embryos or larvae and pooled. Standard methods for protein recovery and Western analyses were utilized. Detailed descriptions of these methods and the specificity of all antibodies used for Western analyses are presented in Text S1. Primary antibodies used were rabbit polyclonal anti-Phospho-Chk1 (Ser345), Phospho-Chk2 (Ser33, Thr68), Chk2 and rabbit polyclonal anti-γH2AX (Ser 139) (Cell Signaling Technology, Danvers, MA) and mouse mAb414 (Covance, Berkeley, CA), rabbit polyclonal Mcm4 (Bethyl Laboratories, Montgomery TX), Mcm2 and Mcm3 (BD Pharmingen, San Jose CA). All blots are representative of at least triplicate experiments.

### Treatment with Replication Inhibitors, Checkpoint Inhibitors, and γ-Radiation

Wild type and sibling *flo* larvae (∼80 hpf) were treated with Chk2 inhibitor, ATM inhibitor, caffeine, hydroxyurea, camptothecin and cisplatin at the doses and times indicated in Table 1. All reagents were purchased from Sigma Aldrich except as listed. For UV irradiation, wild type and *flo* larvae in embryo media were placed 12 cm from a Sankyo Denki UV germicidal bulb (15W) for 10 minutes. γ-irradiation of wild type and *flo* larvae were irradiated as previously described [70]. Additional information regarding treatment with replication inhibitors is presented in Text S1.

## Supporting Information

Figure S1 Combined physical and genetic map of the *flo* locus. A physical contig comprising five BAC clones spanned markers that defined the critical region surrounding the *flo* locus (markers (TAAA)7 and (GAT)6). The number of mutant larvae (within a total of 2629 analyzed) that were recombinant for each marker is listed. Zero mutants were recombinant for the (GAT)6 marker located within the coding region of the *elys* gene. This marker is located 3079 bp from the *flo* mutation.
(80 KB TIF)

Figure S2 Mouse-zebrafish Elys protein alignment. Amino acid alignment of the mouse and zebrafish Elys protein showing the following protein domains: NES, nuclear export signal; WD-40 repeats; coiled-coiled domain; AT hook domain; NLS-nuclear localization signal. The arginine residue (R) targeted by the *flo^ti262c^* mutation is highlighted in light blue.
(5.3 MB PDF)

Figure S3 Reduced *elys* expression in *flo* larvae. Results from real time quantitative PCR amplification of *elys* cDNA fragments. These data show reduced *elys* expression in 5 dpf *flo* vs. sibling wild type larvae. The Elys-1 primers are located in exons 5/6. The Elys-2 primers are located in exons 22/23. The *flo* mutation is located in *elys* exon 30. Reduced *elys* expression in *flo* larvae is consistent with non-sense codon initiated mRNA decay induced by the *flo^ti262c^* mutation (23).
(166 KB TIF)

Figure S4 Morpholino induced *elys* cDNA truncation. (A) Ethidium bromide stained agarose gel showing 1 kb truncation of an *elys* cDNA fragment amplified from 1 dpf and 2 dpf wild type embryos that had been injected with the *elys* exo*n* 30 splice junction morpholino. This morpholino targets genomic sequence at the intron 29/exon 30 splice acceptor. Successful targeting induces deletion of exon 30, as revealed by sequence analysis (B) of the 0.6 kb fragment.
(434 KB TIF)

Figure S5 Nuclear pore defects in early *flo* mutants. (A-D) Histological sections of *flo* mutants and wild type siblings immunostained with anti-FG Nup antibody (mAb414) showing nuclear pore defects in the 36 hpf *flo* retina (A,B) and 48 hpf intestine (C,D). Blue, Dapi stained nuclei.
(1.5 MB TIF)

Figure S6 DNA damage response activation in *flo* mutants. (A,B) Quantitative PCR reveals increased *p53*, *p21*, and *mdm2* expression in *flo* mutants. Note that *tp53* knockdown in *flo* (B) abrogates increased *p21* and *mdm2* expression. (C) Western blot showing comparable levels of phospho-Chk2 in the *flo* and wild type eye (48 hpf). (D) Western blot showing native and phospho-Mcm4 (*) in the zebrafish wild type (wt), *flo* intestines before and after γ-irradiation (30 Gy). Note that there is very little native Mcm4 in wt and *flo* following γ-irradiation (lanes 2 and 3). Phosphatase treatment (λ) of the wt sample from lane 2 dephosphorylates nearly all of the phospho-Mcm4 protein such that only native Mcm4 is present in the sample. (E) Confirmatory Western blot showing reduced chromatin bound Mcm2 in the intestine of 84 hpf *flo* larvae compared with sibling wt larvae. Far right lane labeled “A/G” shows undetectable levels of Mcm2 and Histone 3 recovered from Ig fraction of the wild type intestinal protein prep prior to anti-histone immunoprecipitation. Presumptive phospho-Mcm2 bands on this gel are denoted by the asterisk (*). (F) Western blot showing reduced Mcm2 in 96 hpf *flo* larvae, but normal levels in 96 hpf *slj* larvae compared with control wild type larvae. (G) Western blot showing inhibition of Chk2 phosphorylation in *flo* larvae treated with the Chk2 inhibitor. (H) Western blot showing specificity of the anti-phospho Chk2 antibody. Phospho-Chk2 levels are elevated in 84 hpf *flo* larvae but are reduced when injected with splice morpholinos (splice, two independent sets of injections shown) or morpholino designed against the Chk2 translation initiation site (atg) but not larvae injected with vehicle control (cntrl). (I) Western blot showing specificity of the anti-phospho Chk1 antibody: abundant phospho-Chk1 is present in irradiated Hela cells and the non-irradiated 96 hpf *flo* intestine, but reduced levels are present in the intestine of 96 hpf *flo* treated with the ATR inhibitor caffeine (10 uM; 15 uM beginning at 84 hpf); the intestine of 96 hpf *flo* larvae treated with caffeine (10 uM) and a commercially available ATM inhibitor (Sigma Aldrich; 12 uM); but not in the intestine of 96 hpf *flo* larvae treated with ATM inhibitor alone (12 uM beginning at 84 hpf). His3, anti-Histone 3; A/G, IG fraction recovered following protein A/G precipitation.
(1.1 MB TIF)

Table S1 Reduced cell proliferation in the *flo* intestine. Percentage of S-phase cells is reduced in the 75 hpf *flo* intestine vs. wild type siblings as determined by BrdU immunohistochemistry. The percentage of phospho-Histone H3 cells is comparable in *flo* and wild type larvae. These data are consistent with G1 arrest.
(33 KB PDF)

Table S2 Reduced cell proliferation in the *flo* retina. Data derived from FACS of freshly dissociated retinal epithelial cells from *flo* and sibling wild type larvae. Sorting performed as described in Methods. FACS data is consistent with G1 arrest of rapidly proliferating retinal epithelial cells.
(26 KB PDF)

Text S1 Supplementary data.
(47 KB DOC)
