# Supplementary figures and images for: Mutation of the Zebrafish Nucleoporin elys Sensitizes Tissue Progenitors to Replication Stress
Source: PLoS Genet. 2008 Oct 31;4(10):e1000240. doi: 10.1371/journal.pgen.1000240 (PMC2570612; doi:10.1371/journal.pgen.1000240)

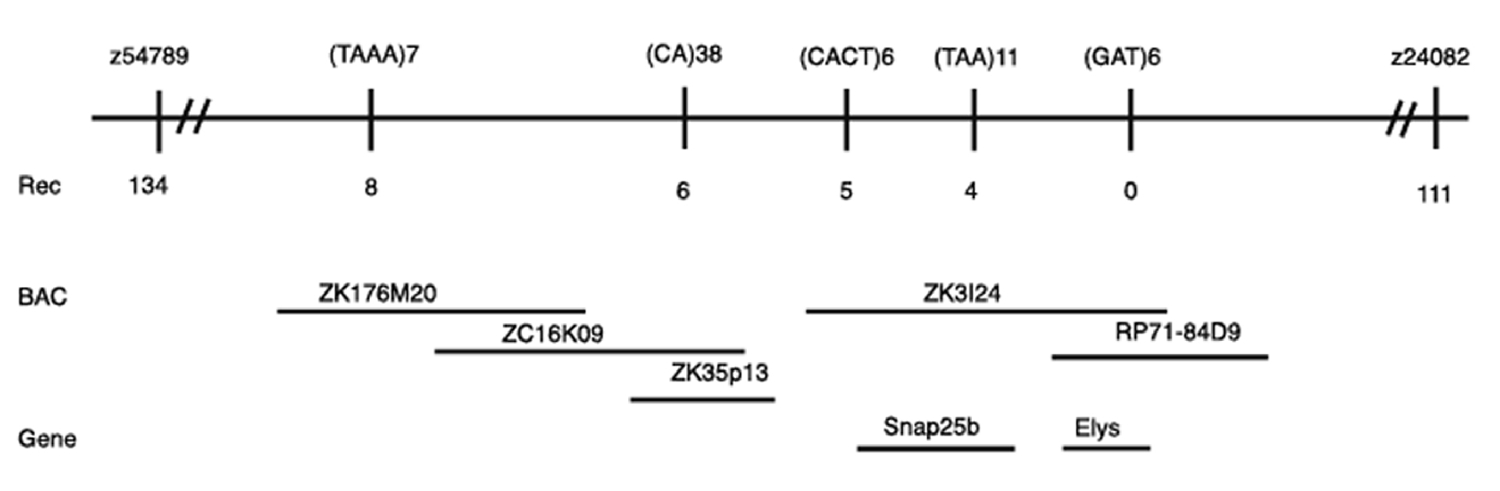

Supplement: Figure S1 — Combined physical and genetic map of the flo locus. A physical contig comprising five BAC clones spanned markers that defined the critical region surrounding the flo locus (markers (TAAA)7 and (GAT)6). The number of mutant larvae (within a total of 2629 analyzed) that were recombinant for each marker is listed. Zero mutants were recombinant for the (GAT)6 marker located within the coding region of the elys gene. This marker is located 3079 bp from the flo mutation. (80 KB TIF) [file pgen.1000240.s001.tif]

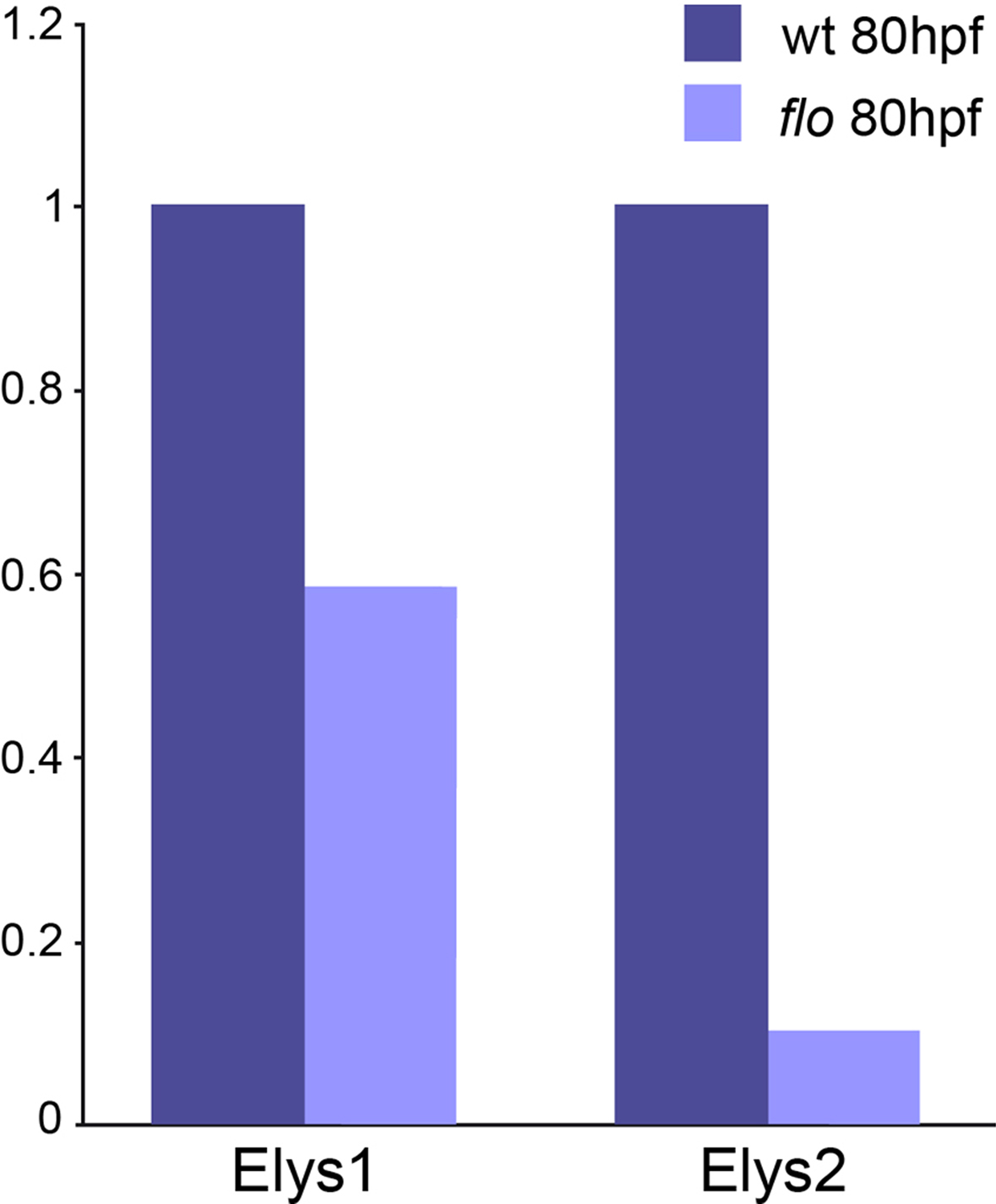

Supplement: Figure S3 — Reduced elys expression in flo larvae. Results from real time quantitative PCR amplification of elys cDNA fragments. These data show reduced elys expression in 5 dpf flo vs. sibling wild type larvae. The Elys-1 primers are located in exons 5/6. The Elys-2 primers are located in exons 22/23. The flo mutation is located in elys exon 30. Reduced elys expression in flo larvae is consistent with non-sense codon initiated mRNA decay induced by the floti262c mutation (23). (166 KB TIF) [file pgen.1000240.s003.tif]

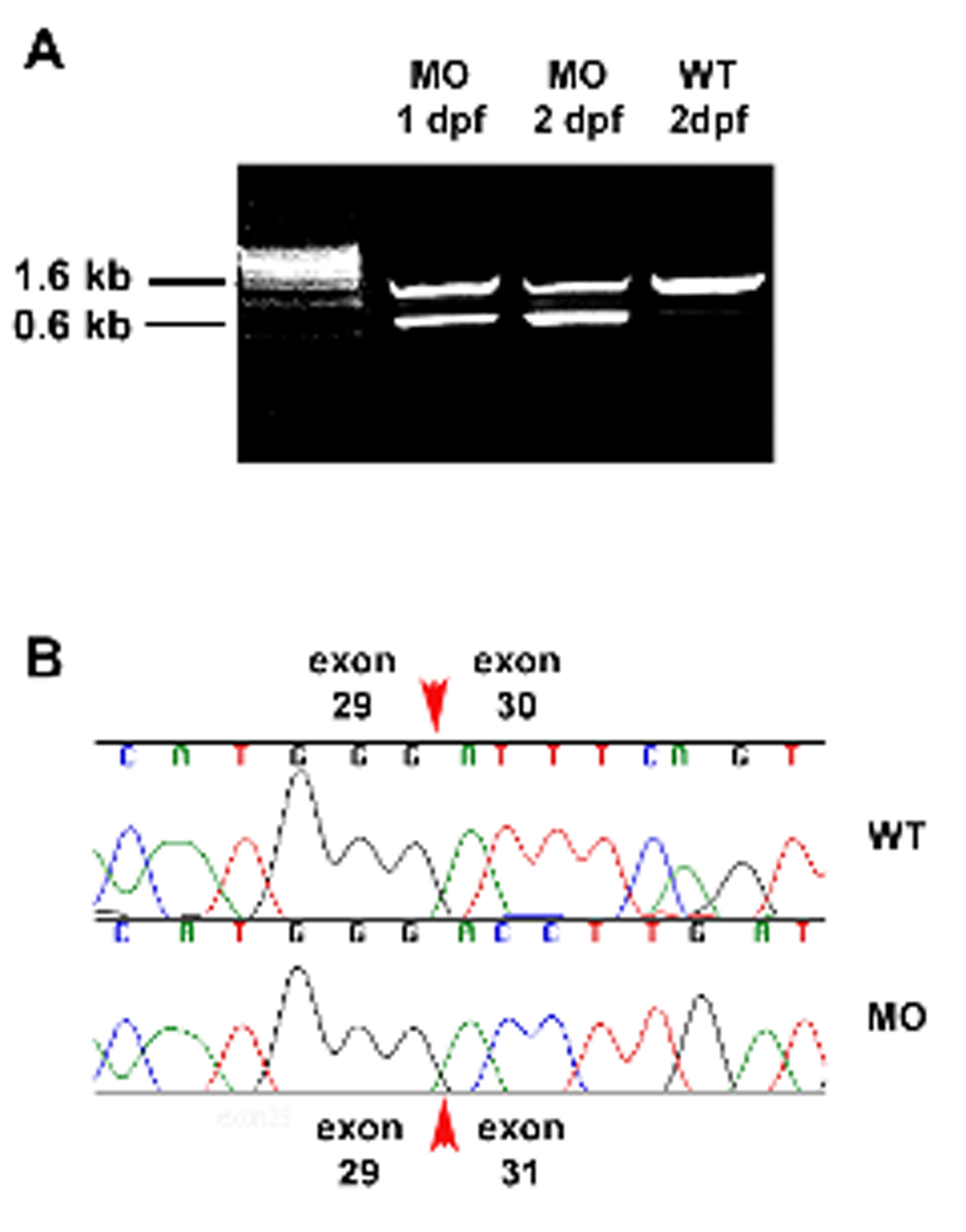

Supplement: Figure S4 — Morpholino induced elys cDNA truncation. (A) Ethidium bromide stained agarose gel showing 1 kb truncation of an elys cDNA fragment amplified from 1 dpf and 2 dpf wild type embryos that had been injected with the elys exon 30 splice junction morpholino. This morpholino targets genomic sequence at the intron 29/exon 30 splice acceptor. Successful targeting induces deletion of exon 30, as revealed by sequence analysis (B) of the 0.6 kb fragment. (434 KB TIF) [file pgen.1000240.s004.tif]

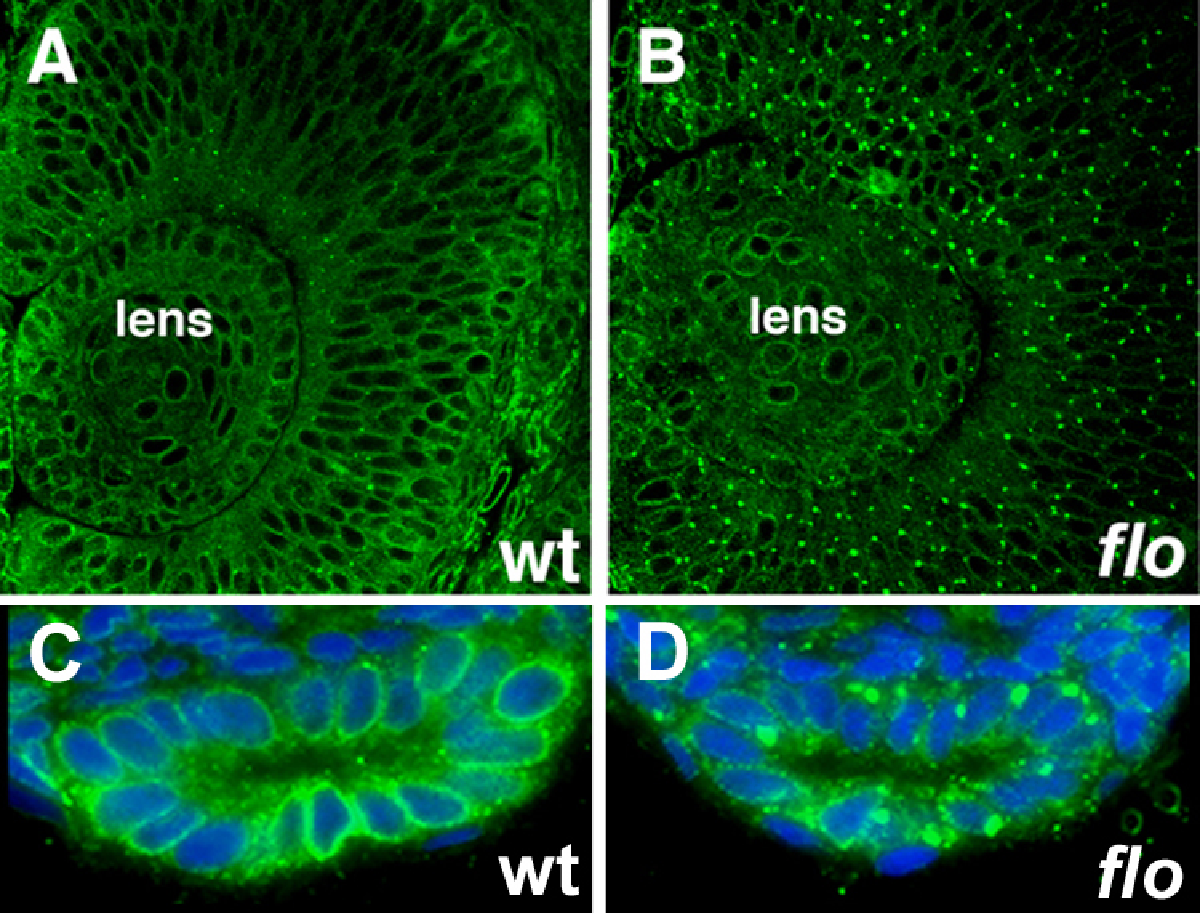

Supplement: Figure S5 — Nuclear pore defects in early flo mutants. (A-D) Histological sections of flo mutants and wild type siblings immunostained with anti-FG Nup antibody (mAb414) showing nuclear pore defects in the 36 hpf flo retina (A,B) and 48 hpf intestine (C,D). Blue, Dapi stained nuclei. (1.5 MB TIF) [file pgen.1000240.s005.tif]

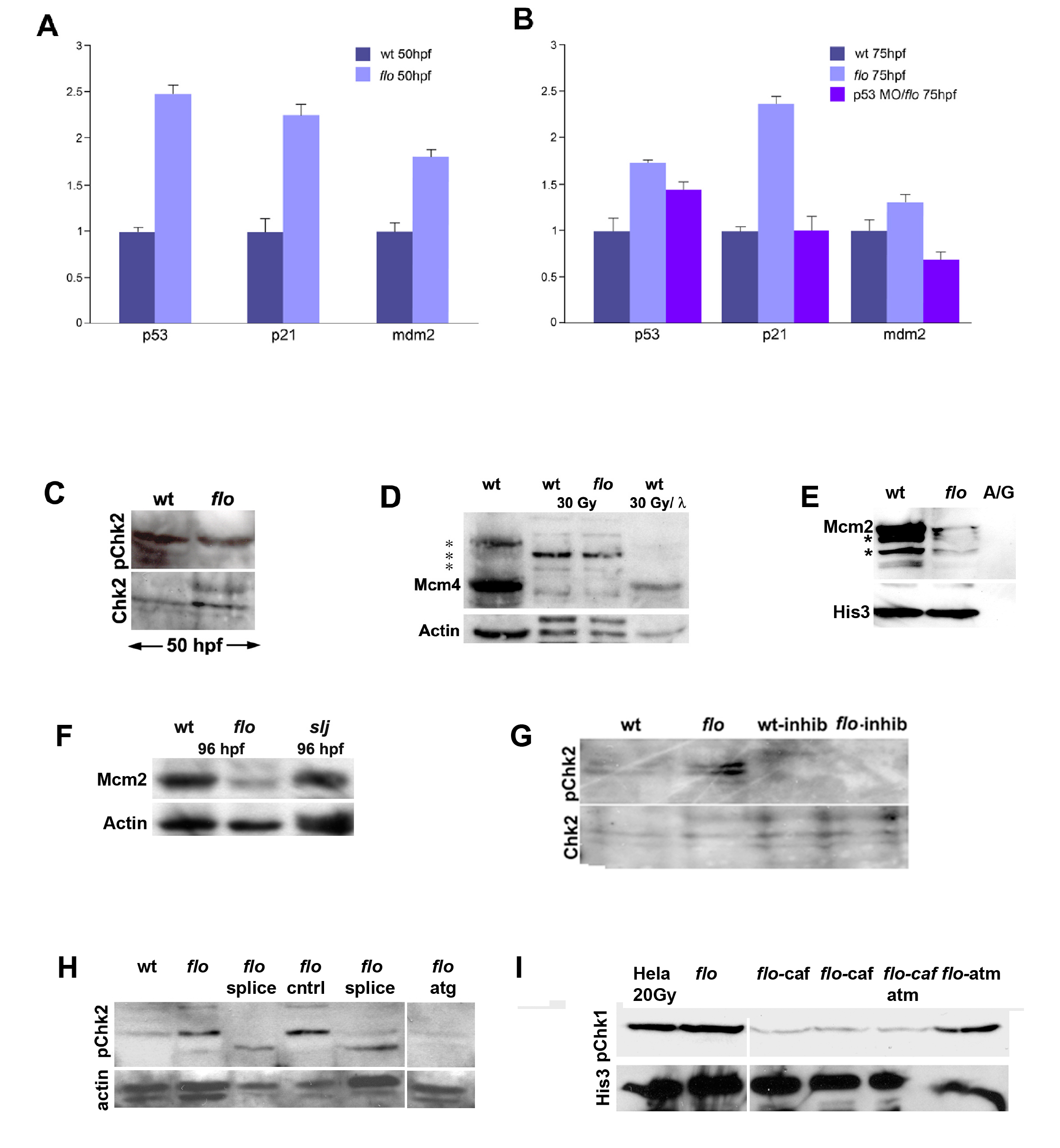

Supplement: Figure S6 — DNA damage response activation in flo mutants. (A,B) Quantitative PCR reveals increased p53, p21, and mdm2 expression in flo mutants. Note that tp53 knockdown in flo (B) abrogates increased p21 and mdm2 expression. (C) Western blot showing comparable levels of phospho-Chk2 in the flo and wild type eye (48 hpf). (D) Western blot showing native and phospho-Mcm4 (*) in the zebrafish wild type (wt), flo intestines before and after γ-irradiation (30 Gy). Note that there is very little native Mcm4 in wt and flo following γ-irradiation (lanes 2 and 3). Phosphatase treatment (λ) of the wt sample from lane 2 dephosphorylates nearly all of the phospho-Mcm4 protein such that only native Mcm4 is present in the sample. (E) Confirmatory Western blot showing reduced chromatin bound Mcm2 in the intestine of 84 hpf flo larvae compared with sibling wt larvae. Far right lane labeled “A/G” shows undetectable levels of Mcm2 and Histone 3 recovered from Ig fraction of the wild type intestinal protein prep prior to anti-histone immunoprecipitation. Presumptive phospho-Mcm2 bands on this gel are denoted by the asterisk (*). (F) Western blot showing reduced Mcm2 in 96 hpf flo larvae, but normal levels in 96 hpf slj larvae compared with control wild type larvae. (G) Western blot showing inhibition of Chk2 phosphorylation in flo larvae treated with the Chk2 inhibitor. (H) Western blot showing specificity of the anti-phospho Chk2 antibody. Phospho-Chk2 levels are elevated in 84 hpf flo larvae but are reduced when injected with splice morpholinos (splice, two independent sets of injections shown) or morpholino designed against the Chk2 translation initiation site (atg) but not larvae injected with vehicle control (cntrl). (I) Western blot showing specificity of the anti-phospho Chk1 antibody: abundant phospho-Chk1 is present in irradiated Hela cells and the non-irradiated 96 hpf flo intestine, but reduced levels are present in the intestine of 96 hpf flo treated with the ATR inhibitor caffeine [file pgen.1000240.s006.tif]
