# Supplementary material for: Mutation of the Zebrafish Nucleoporin elys Sensitizes Tissue Progenitors to Replication Stress
Source: PLoS Genet. 2008 Oct 31;4(10):e1000240. doi: 10.1371/journal.pgen.1000240 (PMC2570612; doi:10.1371/journal.pgen.1000240)
Supplement: Figure S2 — Mouse-zebrafish Elys protein alignment. Amino acid alignment of the mouse and zebrafish Elys protein showing the following protein domains: NES, nuclear export signal; WD-40 repeats; coiled-coiled domain; AT hook domain; NLS-nuclear localization signal. The arginine residue (R) targeted by the floti262c mutation is highlighted in light blue. (5.3 MB PDF) [file pgen.1000240.s002.pdf]

```
zElys          MRDLAAQVTSSLLRFPEVTIDALGEDEVTLDSVLHGKFTVGKSGLAWLACGPQLEVVHAV
mElys          MQDLTAQVTSDLLHFPEVTIEALGEDEITLESVLRGKFAAGKNGLACLACGPQLEVVNSL
               *:**:*****.**:******:******:**:***:***:.**.*** **********:::
                                                               NES-1
zElys          SGERFSAYHFSGVSEHPPTVLAVRDFCWLKRTGLLIGLEEAEGSMLCLYDLGISRVVKAV
mElys          TGERLSAYRFSGVNEQPPVVLAVKEFSWHKRTGLLIGLEEADGSVLCLYDLGISRVVKAV
               :***:***:****.*:**.****::*.* ************:**:***************
                                                              NES-2
zElys          VIPGRITAIEPLVSYGGASASTQHLHQSLRWLFGVAAVVTDIGHVLLVDLCLDDLSCSQS
mElys          VLPGRVTAIEPIINHGGASASTQHLHPSLRWLFGVAAVVTDVGQILLIDLCLDDLSCSQN
               *:***:*****::.:*********** **************:*::**:***********.
                                                                    WD-1
zElys          ELEASDLEVVNKSPSEIPRLREGVTQQGRHLCLQLSSPTGIGATALQYIQRTNQLAVGFS
mElys          EVEASDLEVITGIPAEVPHIRERVMREGRHLCFQLVSPLGVAISTLSYINRTNQLAVGFS
               *:*******:.  *:*:*::** * ::*****:** ** *:. ::*.**:**********

zElys          DGNLQLWNMKSLKKEYHSQLEGGRVPVYAFTFQEPENDPRNCCYLWAVQSAQDLEGDVVS
mElys          DGYLALWNMKSMKREYYTQLEGGRVPVHAVAFQEPENDPRNCCYLWAVQSTQDSEGDVLS
               ** * ******:*:**::*********:*.:*******************:** ****:*
               NES-3
zElys          LHLLQLAFGERKCLASGKIIYEGLEYCXERYSQDLNGTSFPLRAHATNTRLISCQTIEKF
mElys          LHLLQLAFGDRKCLASGQILYEGLEYCEERYTLDLAGGTFPLRGQTSNTKLLGCQSIERF
               *********:*******:*:******* ***: ** * :****.:::**:*:.**:**:*

zElys          RHHPDRXDSMNEVASPDTSVSVFSWQVKSYGQGQPSTFIGVFDINRWYHAQMPDSLRTGE
mElys          PSHGDREESMREALSPDTSVSVFTWQVNIYGQGKPSVYLGLFDINRWYHAQMPDSLRSGE
                 * ** :**.*. *********:***: ****:**.::*:*****************:**

zElys          SLRNCPYLAVWSLDCVIEMTAPCALLDILVHERSLSRGLPYTCPPPEQFFNPTTYNFDGS
mElys          SLHNCSYFALWSLDSVVSRTSPHHILDILVHERSLNRGVPPSYPPPEQFFNPSTFNFDAT
               **:**.*:*:****.*:. *:*  :**********.**:* : *********:*:***.:

zElys          CLFNAGIVHFTCSGYQKETLCYLKKVAPSLGDSISNGYSRCLMSGLLSSRLADVQPSSLS
mElys          CLLDSGVIHVTCAGFQKETLTFLKKSGPTLNEVIPDSYNRCLVAGLLSPRLIDIQPSSLS
               **:::*::*.**:*:***** :*** .*:*.: *.:.*.***::****.** *:******

zElys          QEEQLDAILCTAVETSSLGLITGCXRQWTTEEQPGSAVNLRYILEWAWNKVVHTKEELDE
mElys          QEEQLEAILSAAIQTSSLGLLTGYIRTWIIEEQPNSAANLRFVLEWTWNKVVLTKEEFDR
               *****:***.:*::******:**  * *  ****.**.***::***:***** ****:*.

zElys          ICAPLFDSSANFTDSQTLQLLQHSQRLLANLSTIFHCLLNEAQELTQKGLIGLMNKCMVS
mElys          LCVPLFDGSCRFIDPQTIQSIQQCHLLLSNLSTVLSCFAMEAQGITERGLVDLSNKHMVT
               :*.****.*..* *.**:* :*:.: **:****:: *:  *** :*::**:.* ** **:

zElys          SLVSQYAQVVLWFCRTGLLPEGFDDDDVLQLSRPFYSHSIIKNYYIGQREELRRMAKDKW
mElys          QLLCQYAHMVLWFCHSGLLPEGLD--DALQLSRLRYNYPVIQNYYTSRRQKSERSPRGKW
               .*:.***::*****::******:*  *.*****  *.:.:*:*** .:*:: .* .:.**

zElys          CADCLMIDGLVSQCGERLSELWKRDEGGTGHYPPPSLHALLDIYLLENIEESAKHAIVIY
mElys          NHDCLMIDGLVSQLGDEVEKLWKRDEGGTGRYPPASIHALLDIYLLDNITEASKHAITIY
                 *********** *:.:.:**********:***.*:*********:** *::****.**

zElys          LLLDVMYSFPNKSGASVESFPTAFAIPIGLVKLVQGLWLLDHHDHESSLELLLHPATSHS
mElys          LLLDIMYSFPNKTDTPIESFPTAFAISWGQVKLVQGFWLLDHNDYENGLDLLFHPVTAKP
               ****:*******:.:.:*********. * ******:*****:*:*..*:**:**.*::.
                                                              WD-2
zElys          LWSWQHERVLQALMCQQKHSIALRYLHVMKPPLCTTSQAKLCLSVFLHNRCIVEAWALLR
mElys          AS-WQHSKIIEAFMSQGEHKQALRYLQTMKPTVSSSNEVILHLTVLLFNRCMVEAWNLLR
                  ***.::::*:*.* :*. *****:.***.:.::.:. * *:*:*.***:**** ***

zElys          QHCNKLNTEEVMRFLFETCHELGLMKELLKLPLGLAEQECLQRFLQSNGGFQNRELLMVH
mElys          QNSNRVNIEELLKHAYEVCQEMGLMEDLLKLPFTNTEQECLVKFLQSSTSVENHEFLLVH
               *:.*::* **:::. :*.*:*:***::*****:  :***** :****. ..:*:*:*:**
```

```
zElys           YLQQANYVPALQLNQTLKLNLAADRDPKMKERSNTRNSILNQYGKVLPRVQRKLAIERAK
mElys           HLQRANYISALKLNQILKNNLMSDRDPRLRERSVTRNSILDQYGKILPRVQRKLAVERAK
                :**:***:.**:*** ** ** :****:::*** ******:****:*********:****

zElys           PYQHPT--LIHRVKRPQPLSTVTKRSASENVMTRAAFINNVYSKIEEVWAGNNTTPESSP
mElys           PYHLSTSSVFHEVSRPKPLSAFPKKAITGTVLTRSTFISNVLSKIGEVWASHEPRNGVSL
                **: .*  ::*.*.**:***:..*:: : .*:**::**.** *** ****.::.    *

zElys           FKSPKESEMQVPSRHPSSPDLPEAFVGTPINKLTKRMSRLLDLVVQPSSQNSPESSNMPG
mElys           FNSPKTEQPSPVVHSFPHPELPEAFVGTPISNTSQRISRLLDLVVHPVPQPSQCLEFIQQ
                *:*** .: .   :  . *:**********.: ::*:********:* .* *   . :

zElys           TPTRSITSWAGP------------KSISKAPELSLLQTPQVVKRARALASGPVFS---AF
mElys           SPTRSPLCLLSSSLPLSSQFKRPHQNTSRPSELLLLETPLIVKKAKSLALSATSSGFAEF
                :****  .  ..            :. *:..** **:** :**:*::** ... *    *

zElys           TPQSILRSSLRPTPVATPSASPGRSITPPLRPKESRITFIEDADSPEAPKGSVHWNNGIS
mElys           TPPSILRSGLRTTPLASPSLSPGRSLTPPFRVKETRISFMEEG-------MNTHWTDRAT
                ** *****.**.**:*:** *****:***:* **:**:*:*:.        ..**.:  :

zElys           VNREHNTPKRSSPPAKASIGVWSEHSDAEEEDVDMKMTCMPSLGEQENPDVESETGSSIK
mElys           DDRNTKAFVSTS-----------------FHKCGLPAETEWMKTSDKNTYFPLDVPAKGP
                 :*: ::   :*                  .. .:         .::*. .  :. :.

zElys           EIPLAESTKVLPTLVRRPSLGQEASLVSNQSD-TTLEFHDAPAPEDLLITLKEQQANNSV
mElys           QKVVAESLATHSGRLEKLDVSKEDSTASTRSDQTSLEYHDAPSPEDLEGAVFVSPK----
                :  :***  . .  :.: .:.:* * .*.:** *:**:****:****  ::  .
                                                    coiled-coil region
zElys           DREVTVRLPNTFEEHTAPVERNTTVDLPSLMLPEKEKDNSRGDLSNAERSLGIQEAQNEE
mElys           -------------PASSSTELTTNSTLQTERDNDKDAFKSEGTPSPVKKQIGTGDAAVEA
                               ::..* .*.  * :    :*:  :*.*  * .::.:*  :*  *

zElys           ECVNITNDVVENVSEEQLKGISEAQVLKIDVVIKPELVVPVQEPSSESVPEMSEHLDTRD
mElys           FSELSRLDPVERAEAS--------------------FGVSSVCEGETSTSNSKTSVLDGIV
                 .     * **... .                     * .* * .:.:  . :. **

zElys           PTEAEESLNTSSFFVEALSVPPPLEPLNQSMEENAAYNTIDQPPVVKVSEAASEKAVSEA
mElys           PIESRTSILT----------------------------------------ADHKESVANT
                * *:. *: *                                        *  :::*:::

zElys           SVSKSSMGELVNHEQVTIEPEINEQCQEESMETSDLDAFVEQHLFGPDLTQSNIRESSEK
mElys           VADVESSGSTSSKCPVTSERSLGQKLTLNLKEDEIEAHVPKENVGLPEESPRISAAPSDT
                 .. .* *.  .:  ** * .:.::   :  * .    . ::::  *: :      .*:.

zElys           SLKSDPVCDIKDYQTTSENAADLKTQTSETGTSESRSVVSLNDSDELSSAESAEDSDDEE
mElys           HEIHLIGCENLEVQNSEEEAKNLSFDELYPLGAEKLEYNLSTIEQQFCDLPDDKDSAECD
                       *:  : *.:.*:* :*. :   .  :*. .    . .:::..  . :** : :

zElys           SEEESGDEKVEDSGSEVEIIDEIQGNGRSNHQRPSTLYVEELPSHGQYFQDQANAVLSLI
mElys           AAEVDGELFVAQSN--------------------FTLILEGEEGEAEASDSAAPNMLPKA
                : * .*:  * :*.                     ** :*   ...:  :. *  :*.

zElys           TPEAQLKVLDTDIPEEEGEVVMVGLGPADLDEEDGMCYTELRPSTTLLVPVELADQQQTH
mElys           TKEKPVCHREPHNQER------VTDLPSAVTADQESHKVETLPYVPEPVKVAIAENLLDV
                * *  :   :..  *.      *   *: :  ::    .*  * ..  * * :*::

zElys           LIDRGTMETLSGAPEDSEGFTLMLDPDVEELPDASELDHNAVLLPLHEPEEHQDMENKPT
mElys           IKDTRSKEATPVAAGEAG-------------------DEDGAVIVSKAAHSSRLTNSTPK
                : *  : *: . *. ::                    *.:..::  : ... :  :..*.
                                                                NLS
zElys           TNSEVVILEHTFDVPDNKLEEVNDLVSDIEPVVSGSSKIEEQPAEVLQTVEQIITPEPET
mElys           TVKEPRAETVNTSQNDDMVSSRTLTRRQHALSLNVTSEQEPSAVATPKKRTRKIKETPES
                * .*      . .  *: :.. .    :    :. :*: * ... . :.  :  *.  **:
                                      NLS
zElys           CLSNGDGPDSGPSKMQDAPHTPRSVKVFETPSPENALPLELLLEKDNEVSVLDTGVSAIA
mElys           SERTCSDLKVAPENQLTAQSPPAPRRGKKKDVSQGTLPSSGAVE----------------
                .  . .. . .*.:   *  .* . :  :.  .:.:** .  :*
```

```
                                                              NLS
zElys           KDVAPANQIDTTPSLAEEVEELNTTKQAARPKSPGRPKSPGRPKSPARTKVERPGKDTEE
mElys           ----PEPEPQGTPGRLRLRTQPPEPAAEETPSRTKVRLSSVRKGTPRRLKKSVENGQSTE
                    *  : : **.  .   :   .     *. .    *. *  :* * * .  . :: *
                            NLS                             NLS
zElys           IKENLKEVQHSPSRRTRKTVTFPVPNLEADKDLTEEEQMDSQVPSTPRRVTRSGKQLQDC
mElys           ILDDLKGSEAASHDGTVTELRNANLEDTQNMEYKQDEHSDQQLPLKRKRVRER-------
                * ::**  : :.   * . :  .  :   : : .::*: *.*:* . :** .
                                                          NLS    AT-hook
zElys           EVPVTPRRSTRKTDTEQHISENHQDMPSEKVSKPASPARKTPQKATPRKGSRKTRSTAVD
mElys           EVSVSSVTEEPKLDSSQLPLQTGLDVP-----------------ATPRKRGRPRKVVPLE
                **.*:.  .  * *:.*   :.  *:*                 ***** .*  :  ..::

zElys           DDEGDDNDDDDDDKAALTEHIPNEISEIQLRHSTRKSQPKTADRPEAQQEVPVERAQPNK
mElys           ADGGTTGKEQTSPQKKDVPVVRRSTRNTPARNVSTLEKSVLVPNKEAALVVTSKRRPTKK
                 * *  ..:: . :   .  : ..  :   *: :  .:.  . . **   *. :*  .:*
                                                       NLS
zElys           AHSSPSRVTRKSTRGLTLQSFQQDPAEDATTLNQPLRTPPRSRRKTVATTAEKAVNETYV
mElys           SAEESSKDPSAAVSDLAGGAAHTESADRRDGLLAAAALTPSAQG----------------
                : ...*: . :. .*:  : : :.*:    *  .   .* ::
                                                          NLS
zElys           VDDAQLQSVSRRLTRSRHWNQGDGLDKERLDLIPLEVHAESPQADALVERLKNEEASEKN
mElys           -----------TRTRSRRTMLLTDISEPKTEPLFPPPSVKVPKKKSKAENMEAAAQLKEL
                             ****:     .:.: : :     .: *: .: .*.::     ::
                                                              NLS
zElys           VSVVTEIIRAKRRTTRSAAPHMNETQTIGFEESKLISEEIEKHESKSLASARRTRASKLS
mElys           VSDLSSQFVVSPPALRTRQKSISNTSKLLGELESDPKPLEIIEQKPKRSRTVKTRASRNT
                ** ::. : ..  : *:    :.:*..:  * ..  .     .:. . : : :****: :

zElys           ENSASVDDSVAPLAEQRRTRGRKKGSAVPNEVEIPEILPVPQMLSEGGESDLQSKGDNGL
mElys           G-----------------------------------------------------------

                            NLS                                      NLS
zElys           VDDVPVETEVVKPRKKRTTRTKAAPEPPPPVEISFISPFGSPAETISKTEKRTEEKETPS
mElys           ---------------------KGSSWSPPPVEIKLVSPLASPVDEIKTGKPRKTAEIAG-
                                     *.:. .******.::**:.**.: *.. : *.  : :
                            NLS
zElys           LRMNLRRKRMMDAIFPKPVTRRKKL
mElys           --KTLGRGRKKPSSFPKQILRRKML
                   .* * *   : *** : *** *
```

NES: nuclear export signal
NLS: nuclear localization signal
WD: WD repeats
