# Supplementary material for: Mutation of the Zebrafish Nucleoporin elys Sensitizes Tissue Progenitors to Replication Stress
Source: PLoS Genet. 2008 Oct 31;4(10):e1000240. doi: 10.1371/journal.pgen.1000240 (PMC2570612; doi:10.1371/journal.pgen.1000240)
Supplement: Table S1 — Reduced cell proliferation in the flo intestine. Percentage of S-phase cells is reduced in the 75 hpf flo intestine vs. wild type siblings as determined by BrdU immunohistochemistry. The percentage of phospho-Histone H3 cells is comparable in flo and wild type larvae. These data are consistent with G1 arrest. (33 KB PDF) [file pgen.1000240.s007.pdf]

	BrdU	Dapi	%BrdU			PH3	Dapi	%PH3
wt<br>(n=5)	181<br>195<br>204<br>211<br>230	696<br>718<br>777<br>819<br>828			wt = 2<br>larvae	34<br>37	1079<br>690	3.2%<br>5.3%
Av.	204.2	767.6	26.6%					
flo<br>(n=5)	68<br>81<br>99<br>101<br>111	650<br>696<br>707<br>709<br>805			*flo*= 2<br>larvae	30<br>10	677<br>361	4.4%<br>2.7%
Av.	92	713.4	12.8%					
