# Supplementary material for: Mutation of the Zebrafish Nucleoporin elys Sensitizes Tissue Progenitors to Replication Stress
Source: PLoS Genet. 2008 Oct 31;4(10):e1000240. doi: 10.1371/journal.pgen.1000240 (PMC2570612; doi:10.1371/journal.pgen.1000240)
Supplement: Table S2 — Reduced cell proliferation in the flo retina. Data derived from FACS of freshly dissociated retinal epithelial cells from flo and sibling wild type larvae. Sorting performed as described in Methods. FACS data is consistent with G1 arrest of rapidly proliferating retinal epithelial cells. (26 KB PDF) [file pgen.1000240.s008.pdf]

	Cells	G1	S	G2/M
wt = 50 retinae	17738	48.1%	42.7%	9.2%
flo= 50 retinae	15276	66.2%	26.9%	6.9%
