# Supplementary material for: Mutation of the Zebrafish Nucleoporin elys Sensitizes Tissue Progenitors to Replication Stress
Source: PLoS Genet. 2008 Oct 31;4(10):e1000240. doi: 10.1371/journal.pgen.1000240 (PMC2570612; doi:10.1371/journal.pgen.1000240)
Supplement: Text S1 — Supplementary data. (47 KB DOC) [file pgen.1000240.s009.doc]

**Supplementary Data for:**

**Mutation of the zebrafish nucleoporin *elys* sensitizes tissue progenitors to replication stress**

# Gangarao Davuluri^1*^, Weilong Gong^1*^, Shamila Yusuff^1*^, Kristin Lorent^1^, Manimegalai Muthumani^1^, Amy C. Dolan^1^ and Michael Pack^1, 2 #^

# ^1^Departments of Medicine and ^2^Cell and Developmental Biology, University of Pennsylvania School of Medicine, Philadelphia, PA

^*^ These authors contributed equally to this work

^#^ Corresponding author: [mpack@mail.med.upenn.edu](mailto:mpack@mail.med.upenn.edu)

Short title: DNA repair defect in zebrafish *elys* mutants

**Phenotypic identification of *flo* and *flo/tp53* mutants**

Homozygous *flo* larvae are easily distinguished from wild type siblings at 4 dpf. To identify *flo* mutants at earlier time points, embryos were stained with acridine orange to identify apoptotic retinal cells. All 48 hpf and older homozygous *flo* embryos show obvious acridine orange staining in the retina. By comparison, only a small number of acridine orange positive cells were identified in one wild type embryo (over 1000 analyzed).

Acridine orange staining was also used to identify homozygous *flo/tp53* double mutant embryos. Using molecular genotyping, we confirmed that *flo/tp53* double mutants lack retinal acridine orange staining at 48 hpf and 72 hpf. To identify double mutants at later stages, acridine orange negative embryos were raised to 5 dpf; all *flo/tp53* double mutants resembled homozygous *flo* larvae and thus are easily distinguished from wild type siblings that also lack retinal acridine orange staining at earlier stages.

**Genetic mapping and positional cloning of the *flo* locus**

Bulk-segregant analysis originally established linkage of the *flo* locus to the simple sequence repeat (SSR) marker z1490 on zebrafish chromosome 17. High resolution genetic mapping using a total of 2629 *flo* larvae identified a critical region spanning the *flo* locus bounded by the chromosome 17 SSR markers z24082 and z24006 (MGH SSR map; www.zfin.org). This critical interval was narrowed with the identification of a polymorphism in SSR marker z54789. Using the z54789 and z24006 markers as anchors, a chromosomal walk was performed. Ultimately, novel SSR markers were identified in the following BAC clones (Supplemental Figure 1): zK176M20 – marker (TAA)7; zC16K09 – markers(CA)38 and (TAA)11; zK3I24 – markers (CACT)6, (TAA)11 and (GAT)6. Zero of 2629 *flo* larvae were recombinant for the one of the zK3124 markers, (GAT)6. Computational analyses of zK3I24 BAC sequence predicted two genes. One was the zebrafish *synaptosome-associated protein 25b* ortholog (*snap25b*). Sequence analyses did not reveal a mutation in the *flo snap25b* coding region. The second gene had high sequence homology to the mammalian *embryonic large molecule derived from yolk sac* gene, *Elys* (61.4% and 62.8% similarity of amino acids to the human and mouse ELYS gene products, respectively; Supplemental figure 2), suggesting that it is the zebrafish ortholog. Sequence analysis identified a cytosine to thymidine transition in the coding region of the *flo^ti262c^ elys* allele. The mutation converted the codon encoding arginine 1319 (CGA) to a premature stop codon (TGA). Consistent with this, the zero recombinant for marker (GAT)6 was located within exon 34 of the *elys* coding region. Four *flo* larvae were recombinant for the (TAA)11 marker located in intron seven of the *snap25b* gene.

**Primer sequences for genetic mapping, genotyping *flo* embryos, and quantitative PCR**

***Elys sequencing***

Elys-50 TAGTAGTCGGCACGGCTTAACACTG

Elys-702 ACTCTGGTGTAGATGCTGTGTGCTG

Elys-594 TATCTCTCGTGTTGTCAAGGCTGTG

Elys-2326 GCTGTAGCACATCATCATCATCGAA

Elys-2298 AGAAGGCTTCGATGATGATGATGTG

Elys-3966 AACTGAAATCCCATTGTTCCAGTGA

Elys-3906 GAGCCGCATTACCTTCATAGAGGAT

Elys-4783 GAGGCTCTAATGGAGGAGGAACACT

Elys-4688 ATCTTGATACTCGGGACCCAACAG

Elys-5535 ACACATACCATCTTCCTCATCCAGA

Elys-5427 ACCTGAGGCACAGCTGAAAGTTCTA

Elys-6415 TGTCTGCCTCAAGGTTAGGTACAGG

Elys-6209 TTAACACAACCAAACAAGCAGCAAG

Elys-7185 AAGAGCATCAGCTTGAGGAGATTCA

Elys-7149 GGAAGTACACGCTGAATCTCCTCAA

Elys-7860 TCCAGGCAGAGTTCATAATCAAAGC

***flo* genotyping and mapping primers**

AfI lll-F: CACAAAAGTGCTTCCCACTCACGTG

AfI lll-R: GAGAAGGCAAGTCCACAGTTGTGTTC

Hph I-F: CACAAAAGTGCTTCCCACTCTCGGG

Hph I-R: CTCGGGAGCAGGAGCATCATGAAA

TAA11-F GGATGAAAGTCAGAATTGCGTA

TAA11-R AAATTCAGGATGTTTAAATGCAA

GAT6-F ATCAAAGCCTGCAAGTCCTG

GAT6-R GGGATTTTCTGGTGCTGTGT

**Quantitative RT-PCR primers**

Elys-1F: GATGGCAATCTGCAACTCTG

Elys-1R: TGGCTCCTGGAAGGTAAATG

Elys-2F: GAAACTGCCTCTTGGACTGG

Elys-2R: GGAGGTAGTGGACCATGAGC

tp53-F: GTGGCTCTTGCTGGGACAT;

tp53-R: GATGGCTGAGGCTGTTCTTC;

p21-F: TGAGAACTTACTGGCAGCTTCA

p21-R: AGCTGCATTCGTCTCGTAGC

mdm2-F: CAGGAGGAGGAGAAGCAGTG

mdm2-R: AGGGAAAAGCTGTCCGACTT

**Morpholino injections**

The *elys*-exon30 splice donor morpholino induced deletion of 1006 base pairs encoded by *elys* exon 30 (Supplemental Figure 4). Sequence analysis (Supplemental Figure 4) revealed that this deletion generated a frame shift at Elys amino acid 1243 which in turn led to replacement of the C-terminal 1286 amino acids of wild type Elys with fourteen novel amino acids followed by a premature stop codon. The sequences of morpholinos used in this study are:

*elys* -ATG ATGCCTACTGCGCGTTGATGACAAC

*elys* -5UTR ACGTTACTTCAAACACGACTTTCGT

*elys* - exon 30 AAATATAAATTACCTGAGGTTTCCAT

*tp53* - ATG GCGCCATTGCTTTGCAAGAATTG

*tp53* -exon 5 AAAATGTCTGTACTATCTCCATCCG

**Chromatin Isolation**

Both WT and *flo* fish embryos were fixed in 1% formaldehyde for 15 min at room temperature. The intestine was then dissected out and added to cell lysis buffer (5mM PIPES, 85mM KCL, 0.5% NP40, PMSF and Protease inhibitors; 250 ul per 50 larvae). These samples were centrifuged at 5000rpm for 5min at 4°C to pellet the nuclei. The nuclei were resuspended in lysis buffer (50mM Tris-Cl, 10mM EDTA, 1%SDS and protease inhibitors), incubated on ice for 10min and then centrifuged at 14000rpm for 10min at 4°C. Following this, the supernatant was removed and the pellet diluted to 200ul in IP dilution buffer (0.01% SDS, 1.1% Triton X100, 1.2mM EDTA, 16.7mM Tris-Cl, 167mM NaCl). Next, 1ug of rabbit anti-Histone H2 antibody (Upstate Biotechnology or Sigma Aldrich) was added and the mixture incubated overnight at 4°C. Next, 10ul of Protein A/G Agarose (Santa Cruz Biotechnology) was added to the samples and incubated for 1hr at 4°C. The samples were then washed 3 times with PBST and the pellet was dissolved in 50ul of sample buffer. 25ul of samples were run on 10% SDS-PAGE gel for western analysis.

**Western analyses**

Nuclear protein was recovered from embryos and larvae using the following protocol. Dissected tissues or whole embryos were washed in cold PBS and homogenized in 500ul sucrose buffer (300mM Sucrose, 3mM CaCl2, 2mM MgAc, 0.1mM EDTA, 1mM DTT, 0.1mM PMSF, and 1mM Protease mix) with NP-40 by gently pipetting with a 200uL tip. The homogenized embryos or tissues were incubated on ice for 5 minutes to lyse cell membranes and the nuclei were pelleted by centrifugation at 1500xg for 5 minutes. The pelleted nuclei were gently washed in 500uL sucrose buffer without NP-40 and resuspended in 50uL low salt buffer (20mM HEPES pH 7.9, 25% glycerol, 1.5mM MgCl2, 20mM KCl, 0.2mM EDTA, 1mM DTT and 0.1mM PMSF). 0.2X volume of high salt buffer (20mM HEPES pH 7.9, 25% glycerol, 1.5mM MgCl2, 260mM KCl, 0.2mM EDTA, 1mM DTT and 0.1mM PMSF) was slowly added and the suspension was gently mixed. Additional 0.2X high salt buffer was added until 1X volume had been added. These tubes were incubated on the rotary platform at 4°C for 20 minutes and centrifuged at 13000xg for 15 minutes to retain the supernatant (nuclear fraction). Protein levels were quantified by measuring absorption at 320 nM.

For Western blotting, equivalent amounts of protein from nuclear extracts were separated in a 10% SDS–polyacrylamide gel and then electrophoretically transferred to a nitrocellulose membrane using Bio-Rad protein mini gel apparatus (Bio-Rad Laboratories, 1000 Alfred Nobel Drive, Hercules, CA-USA). The membranes were blocked overnight at 4°C in TBS containing 0.1% Tween 20 and 5% skimmed milk. The blots were probed with antibodies listed in the Methods section. Following washes, the blots were incubated with horseradish peroxidase (HRP)–conjugated rabbit anti-rabbit IgG at a 1:5000 dilution for 2hrs. Detection of immunoreactive proteins was performed with an ECL system (Amersham Biosciences Corp., Piscataway, NJ, USA).

The zebrafish Chk2 ortholog is predicted to encode a 503 amino acid protein (Genbank accession number AAK52419). This size correlates with the ~ 55 kD protein recognized by antibodies raised against the mammalian Chk2 protein and an 18 amino acid peptide overlapping the threonine-68 phosphorylation site recognized by the ATM kinase and a peptide overlapping the serine-33 phosphorylation site. Following γ-irradiation , the levels of the peptides recognized by both phospho-Chk2 antibodies increased markedly compared with total Chk2 protein. Morpholinos targeting either the translation initiation codon or the intron 1 – exon 2 splice donor of the zebrafish Chk2 gene abolished detection of total Chk2 protein (not shown) and phospho-Chk2 protein (Ser-33) as shown in supplementary Figure 6G. The zebrafish Chk1 ortholog is predicted to encode a 406 amino acid protein (Genbank BC045904). This size correlates with the ~ 50kD protein recognized by antibodies raised against a 24 amino acid peptide of the mammalian Chk1 protein that spans the serine-345 phosphorylation site. 17 of 24 amino acids within the corresponding region of the zebrafish Chk1 ortholog are identical or similar to the mammalian Chk1 protein. Levels of phospho-Chk1 were markedly reduced following treatment of zebrafish embryos with caffeine, an inhibitor of the ATR kinase that phophorylates mammalian Chk1 (Supplementary Figure 6I). The zebrafish H2AX ortholog is predicted to encode a 142 amino acid protein (Genbank BC046078) that has high sequence homology to mouse H2AX (124 identical amino acids). The serine-139 phosphorylation site is conserved in the zebrafish H2AX protein. Zebrafish and human Mcm2 (Genbank NP_775364), Mcm3 (Genbank NP_997732.1) and Mcm4 (Genebank AAH65958) orthologs have nearly identical molecular weights. The predicted mass of the zebrafish Mcm2, Mcm3 and Mcm4 orthologs is 98.2 kD, 96.4 kD and 90.4 kD, respectively. Strong bands on Western blots corresponding to these molecular weights were identified using the respective antibodies raised against the orthologous human Mcm proteins. Slower migrating bands associated with Mcm3 and Mcm4 proteins were consistently identified. Based on published data (45) (46) and phosphatase treatments (Mcm4; Supplementary Figure 6D), these bands are identified as phospho-proteins. Phospho-Mcm2, a faster migrating band was not consistently identified in our analyses.

**Flow cytometry**

Retinae from fifty 40 hpf wild type and *flo* embryos were recovered by manual dissection. A single cell suspension of the pooled retinae was generated as described (38). Analyses were performed using a BD Biosciences LSR II (San Jose, CA). The pulse area measurement was performed with digital electronics and BD FACSDiVa software to acquire the data. Data analysis was performed using ModFit LT software from Verity Software House (Topsham, ME). A standard diploid model with a manual analysis and AutoLinearity enabled was used.

**DNA replication inhibitor treatment**

In addition to the inhibitor treatments noted in the manuscript text, 84 hpf wild type and *flo* larvae were treated with caffeine (10uM, 15 uM) for 12 hours and evaluated at 96 hpf or 108 hpf. Identical treatment with an ATM inhibitor (12 uM) was also conducted. Lethality associated with inhibitor treatment was defined as severely reduced or absence of cardiac contraction and blood circulation.
